# Oral Mycobiome Alterations in Children with Phenylketonuria: Associations with Dietary Intake and Metabolic Context—A Pilot Study

**DOI:** 10.3390/nu18111764

**Published:** 2026-05-30

**Authors:** Małgorzata Ostrowska, Elwira Komoń-Janczara, Bożena Mikołuć, Katarzyna Iłowiecka, Justyna Jarczak, Justyna Zagórska, Paulina Zambrzycka, Silvia Turroni, Hubert Szczerba

**Affiliations:** 1 Department of Biotechnology, Microbiology and Human Nutrition, University of Life Sciences in Lublin, 20-704 Lublin, Poland; hubert.szczerba@up.edu.pl; 2 Department of Pediatrics, Rheumatology, Immunology and Metabolic Bone Diseases, Medical University of Bialystok, 15-274 Bialystok, Poland; bozenam@mp.pl; 3 Nutrition Clinic, Department of Clinical Dietetics, Medical University of Lublin, 20-093 Lublin, Poland; katarzyna.ilowiecka@umlub.edu.pl; 4 Laboratory of Regenerative Medicine, Preclinical Research and Technology Center, Medical University of Warsaw, 02-097 Warsaw, Poland; justyna.jarczak@wum.edu.pl; 5 Department of Food and Nutrition, Medical University of Lublin, 20-093 Lublin, Poland; justyna.zagorska@umlub.edu.pl; 6 Metabolic Clinic, Department of Pediatrics, Rheumatology, Immunology and Metabolic Bone Diseases, Ludwig Zamenhoff University Children’s Clinical Hospital, Medical University of Bialystok, 15-274 Bialystok, Poland; paulina.zambrzycka@udsk.pl; 7 Unit of Microbiome Science and Biotechnology, Department of Pharmacy and Biotechnology, University of Bologna, 40126 Bologna, Italy; silvia.turroni@unibo.it

**Keywords:** phenylketonuria (PKU), oral mycobiome, fungal community shifts, nutrient–microbiome interactions, metabolic disorders, pediatric nutrition

## Abstract

Background: Phenylketonuria (PKU) is a metabolic disorder requiring a strict low-phenylalanine diet. Oral health impairment, including bacteriome dysbiosis, is common in PKU, yet the mycobiome remains poorly defined. This pilot study aimed to characterise the salivary oral mycobiome of children with PKU compared with controls and to explore associations with dietary intake. Methods: Saliva samples from 18 children, including 8 patients with PKU and 10 age-matched controls, were profiled using internal transcribed spacer (ITS) amplicon sequencing. Alpha/beta diversity, taxonomic composition, diet–fungi correlations, discriminative taxa and LEfSe were analysed. Results: Alpha diversity did not differ significantly between groups after correction for multiple comparisons, although exploratory subgroup analyses suggested lower evenness in PKU children aged <10 years compared with older controls. Beta diversity differed by diagnosis (PERMANOVA: F = 1.7251, *p* = 0.0062) and in the age–diagnosis model (F = 1.8502, *p* = 0.0004). Taxonomic analyses identified nominal differences in several fungal taxa, including *Candida* (*p* = 0.011), *Saccharomycetales_fam_Incertae_sedis* (*p* = 0.011), *Naganishia* (*p* = 0.020), and *Aspergillaceae* (*p* = 0.036) in PKU samples; however, these findings should be interpreted as exploratory because many did not remain significant after FDR correction. Diet–mycobiome analyses identified selected FDR-supported associations, including an inverse relationship between phenylalanine intake and *Naganishia* in PKU. Conclusions: This pilot study suggests preliminary compositional differences in the oral mycobiome of children with PKU that may be related to dietary therapy and metabolic context. These exploratory findings require validation in larger cohorts with detailed oral health assessment and control of confounders.

## 1. Introduction

In recent years, interest in the oral microbiome and its relationship with health and disease has increased, supported by the ease of sampling (compared to other human microbiomes) and advances in high-throughput sequencing for microbial profiling [1,2]. The oral microbiome comprises bacterial, fungal (the mycobiome) and viral communities that interact with each other and with host factors, including diet and oral hygiene. Such interactions have been previously described in association with dental caries [3,4], type II diabetes [5], obesity [6], and phenylketonuria (PKU) [3,7]. Although PKU is primarily a metabolic disorder of phenylalanine metabolism, its lifelong dietary management may alter the oral ecosystem by changing nutrient availability, substrate exposure, and microbial ecology [3,8,9].

PKU (OMIM 261600) is an autosomal recessive disorder caused by pathogenic variants in the phenylalanine hydroxylase gene (PAH; EC 1.14.16.1), leading to elevated blood phenylalanine (Phe) concentrations. Without timely and sustained treatment, chronic hyperphenylalaninaemia contributes to Phe accumulation in the central nervous system and is associated with adverse neurocognitive outcomes [10]. The estimated global prevalence is 1 in 23,930 live births, affecting 450,000 patients [11]. The prevalence of PKU is as follows: 1 in 2700 in Italy, 1 in 5360 in Germany, 1 in 8309 in Poland, 1 in 25,000 in the United States, and 1 in 125,000 in Japan [11]. PKU is identified through newborn screening and requires lifelong management, with dietary therapy remaining the cornerstone of treatment. This approach is based on an individualised low-Phe diet tailored to the patient’s Phe tolerance, defined as the amount of protein (expressed as mg Phe/kg/day) that maintains blood Phe concentrations within recommended therapeutic ranges [10]. Additional approaches may include large neutral amino acids (LNAAs), glycomacropeptides (GMPs) [12], synthetic biotics (SYNB1618, SYNB1934) [13], and pharmacological therapies (sepiapterin and pegvaliase in selected patients) [14,15]. Blood Phe remains the key biomarker for monitoring metabolic control, with European recommendations defining age-dependent target ranges: 120–360 µmol/L for children up to 12 years and 120–600 µmol/L for individuals over 12 years [10]. Early initiation of dietary treatment shortly after birth, following newborn screening diagnosis, is essential to prevent neurocognitive impairment in children with PKU [10,16]. Adherence to a lifelong low-phenylalanine diet may be challenging; therefore, adjunctive or alternative therapeutic options are increasingly considered [10]. Dietary treatment requires regular administration of a Phe-free protein substitute, typically providing the majority (up to approximately 80%) of daily protein requirements, combined with a quantitatively controlled intake of natural protein according to its Phe content and the use of specialised low-protein foods. Foods naturally high in protein and Phe are excluded or markedly restricted [10,17].

Oral health is a clinically relevant aspect of PKU management. Children with PKU have been reported to present higher caries experience, increased dental erosion, enamel defects, altered periodontal parameters, and shifts in oral microbial diversity compared with the general population [3,9,18]. Proposed contributing factors include frequent intake of carbohydrate-containing foods and acidic specialised products, alongside challenges in maintaining optimal oral hygiene [3,9]. These observations support the hypothesis that PKU-related dietary patterns may shape oral microbial ecology and contribute to oral disease risk. Importantly, the dietary management of PKU is typically characterised by frequent intake of carbohydrate-rich low-protein foods and specialised nutritional preparations with altered physicochemical properties [19,20]. Such dietary patterns may modify salivary composition, oral pH, and nutrient availability, thereby influencing fungal colonisation and inter-kingdom biofilm formation [21,22,23]. *Candida* species, one of the dominant fungal taxa of the oral cavity, efficiently metabolises carbohydrates and interacts with cariogenic bacteria, potentially contributing to oral dysbiosis and caries development [24,25]. Therefore, investigation of the oral mycobiome may provide additional insight into the relationship between long-term PKU dietary treatment and oral health. Accordingly, oral health guidance includes rinsing with water after protein substitutes, consuming them quickly, limiting sweet foods and drinks to meals, and avoiding feeding bottles beyond 1 year of age [10].

Although most oral microbiome studies focus on bacteria, fungal communities represent an important yet comparatively understudied component of the oral ecosystem. The oral mycobiome is diverse, with *Candida* species commonly detected in saliva and dental plaque. Fungal community structure appears to be closely associated with oral health status; for example, reduced mycobiome diversity has been reported in children with caries compared with caries-free peers [24,26]. As oral fungi, like bacteria, respond to nutrient availability and local physicochemical conditions, diet-related changes in the oral environment may promote fungal dysbiosis and potentially increase susceptibility to conditions such as early childhood caries (ECC) [24,26].

Despite the growing interest in microbiome alterations in PKU, previous research has primarily focused on bacterial populations in the gut [7,27,28,29]. The oral microbiome of individuals with PKU remains poorly characterised [3,7], particularly with regard to fungal communities, despite the known role of the mycobiome in mucosal ecology and oral disease. Considering the role of *Candida* within the oral mycobiome and its ability to thrive under carbohydrate-rich conditions, it is important to determine whether children with PKU have different oral fungal compositions from healthy peers. Understanding PKU-associated changes in the oral mycobiome may clarify disease- and diet-related effects on host physiology and inform more targeted oral health strategies for patients on a PKU diet.

Therefore, the aim of this study was to characterise the oral mycobiome of children with PKU compared with a healthy control group using next-generation sequencing. We hypothesised that long-term dietary treatment for PKU and age would be associated with measurable differences in salivary fungal diversity and community composition, and that specific dietary factors would correlate with mycobiome characteristics. To test this hypothesis, the subjects were categorised into age-matched subgroups, in accordance with recognised standards [30]. In line with previous studies [31], we analysed two age categories: children aged 3–9 years and those aged ≥10 years. We also examined the associations between dietary intake (including macronutrients, micronutrients and vitamins) and the proportions of oral fungi. This work will improve our understanding of how long-term dietary interventions shape the oral ecosystem in metabolic disorders, potentially informing nutritional care and oral disease prevention in PKU.

## 2. Materials and Methods

### 2.1. Subject Cohorts

This preliminary study involved 18 children from the Polish population. Participants were assigned to two groups: 8 patients diagnosed with PKU and 10 controls without PKU. Each group was further stratified into two age categories: children aged <10 years and those aged ≥10 and <18 years. Controls were recruited to achieve approximate group-level matching with the PKU group in terms of age, sex, BMI, and nutritional status. Matching was therefore performed to improve overall comparability between groups rather than as strict one-to-one individual pair matching. Because of the limited availability of eligible participants and the exploratory nature of the study, exact matching for all variables was not always achievable [32]. Participants were recruited through metabolic disease centres across Poland, which coordinated the enrolment of both cohorts. Children were eligible for inclusion in the PKU group if they had been diagnosed with PKU through newborn screening, were under 18 years of age, and had a Phe level measurement available. All children with PKU remained under regular specialist metabolic and dietary care. Dietary management was supervised by metabolic clinicians and dietitians, and PKU-specific medical foods/protein substitutes were reported in dietary diaries when available. However, blood Phe concentrations were not available for every dietary recording period, and detailed longitudinal metabolic control variables (e.g., historical Phe variability, Phe tolerance, duration of dietary adherence) were not consistently available for all participants; therefore, these parameters were not incorporated quantitatively into the present analyses. Children without PKU who were under 18 years old were eligible for inclusion in the control group. In both groups, exclusion criteria included congenital malformations, chronic liver disease, chronic or acute intestinal disease, antibiotic treatment, and probiotic use within 3 months prior to study entry. Each participant completed a comprehensive nutritional questionnaire that collected demographic, anthropometric, and clinical data, including age, sex, height, weight, medical history, medication usage, and lifestyle habits, as well as a three-day food diary and a food frequency questionnaire [32].

This study was conducted in accordance with the Declaration of Helsinki and approved by the Bioethical Commission of the Medical University of Lublin, approval number. KE-0254/279/2017, 23 November 2017, and the Bioethical Commission of the Institute of Rural Health of Lublin, approval n. 1/2023, 8 February 2023. All data were fully anonymised prior to access by the authors. Parents or legal guardians were provided with detailed instructions on weighing and recording meals to ensure accurate dietary assessment.

### 2.2. Sample Collection

Saliva samples collection was performed during the same period as dietary data acquisition. Samples were self-collected at home with the assistance of parents or legal guardians trained in the collection procedure, using GeneFix™ Saliva Microbiome DNA Collector tubes (Iso-helix™, Kent, UK), which contain a stabilising buffer allowing storage at room temperature for up to 1 month. Collection was performed at least 1 h after a meal, with participants instructed to refrain from chewing gum, tooth brushing, and drinking (except for plain water). Approximately 2 mL of saliva was collected. Upon delivery to the department, samples were additionally coded and stored at −20 °C until analysis. To minimise pre-analytical variability, all participants received the same written collection instructions and used the same saliva collection kit containing stabilising buffer. Samples from both PKU and control participants were collected during the same study period and delivered to the laboratory by courier within 5 days of collection. No intentional differences in collection, transport, or storage procedures occurred between study groups.

### 2.3. Evaluation of Nutritional Condition, Food Consumption and Group-Level Comparisons

Dietary intake data were derived from the same cohort previously described in [31], where they were analysed in the context of gut microbiome composition. In the present study, dietary variables were re-analysed in relation to oral mycobiome profiles. Two participants were excluded due to the unavailability of an oral sample. Nutritional status was assessed based on BMI values analysed according to current Polish reference standards, represented as percentile grids for children of both genders aged 3.0–17.5 years [33]. The nutritional composition of consumed foods and meals was evaluated using Aliant software (Cambridge Diagnostics, Warsaw, Poland), a commercially available application integrated with the official Polish food composition database (database version 6.0; software version 61) [34]. Parents or legal guardians of children with PKU completed three-day dietary records, which were then entered into the software by a certified dietitian (KI). These records were used to estimate daily energy and nutrient intake. The results were analysed in relation to the dietary reference intakes established for the Polish population, according to the participants’ age groups. Dietary fibre intake was additionally evaluated against the adequate intake (AI) level [35].

An acceptable deviation from the reference value was established at ±10%. When AI served as the benchmark, intake at or exceeding the AI was deemed sufficient, whereas intake below the AI was regarded as inadequate. The Shapiro–Wilk test was used to assess the normality of the distribution for continuous variables. Based on this, comparisons between two means were performed using either a Student’s *t*-test for normally distributed data or the non-parametric Mann–Whitney U test for data that did not satisfy the assumption of normality. Because the matching procedure was applied at the recruitment stage to improve group-level comparability and did not constitute a strict one-to-one matched-pairs design, comparisons of demographic, anthropometric, and dietary variables were performed using group-level statistical tests rather than paired analyses.

### 2.4. Fungal ITS Amplicon Sequencing

Fungal genomic DNA was isolated from saliva samples using the GeneFiX ™ DNA Isolation Kits (Isohelix ™, Kent, UK) following the manufacturer’s instructions, and stored at −20 °C until molecular analysis. In brief, 100 U (10 µL) of lyticase (A&A Biotechnology, Gdansk, Poland) was added to 1 mL of the dissolved saliva sample. After shaking, the mixture was incubated at 30 °C for 30 min. The subsequent steps were performed in accordance with the manufacturer’s specifications. DNA concentration was determined on a Qubit 4.0 fluorometer (Invitrogen, Waltham, MA, USA), and DNA purity was assessed using a NanoDrop™ 2000 spectrophotometer (Thermo Fisher Scientific, Waltham, MA, USA). Only DNA samples with purity ratios 260/280 and 260/230 > 1.8 and a concentration ≥ 5 ng/µL were used in the study. For each 25 ng DNA sample, PCR was performed using 2X KAPA HiFi HotStart ReadyMix polymerase (Roche Kapa Biosystems, Wilmington, NC, USA), for the target region ITS1–ITS2 using specific primers [36] (with adapter sequences: forward 5′-TCGTCGGCAGCGTCAGATGTGTATAAGAGACAG-[locus-specific sequence] and reverse 5′-GTCTCGTGGGCTCGGAGATGTGTATAAGAGACAG-[locus-specific sequence]) as described in the Fungal Metagenomic Sequencing Demonstrated Protocol (Illumina, San Diego, CA, USA). The following thermal cycling parameters were used: 95 °C for 3 min; 25 cycles of 95 °C for 30 s, 55 °C for 30 s, and 72 °C for 30 s, and a final extension at 72 °C for 5 min. DNA amplicons were purified using Agencourt^®^ AMPure^®^ XP magnetic beads (Beckman Coulter, Brea, CA, USA) following the manufacturer’s instructions. Next, the libraries were double-indexed using the Nextera XT kit according to the manufacturer’s instructions (Illumina). A PCR reaction of 50 μL was performed under the following conditions: 95 °C for 3 min; 8 cycles of 95 °C for 30 s, 55 °C for 30 s, and 72 °C for 30 s, and a final extension at 72 °C for 5 min. The final libraries were cleaned up using Agencourt^®^ AMPure^®^ XP magnetic beads (Beckman Coulter). Libraries were quantified using a Qubit dsDNA BR assay (Invitrogen). The size of the libraries (360–390 bp) was checked using a TapeStation Desktop System (Agilent Technologies, Santa Clara, CA, USA). Libraries were then pooled at equimolar concentrations, ensuring normalisation across the different samples sequenced in the same run. The final concentration was 50 pM. All libraries were sequenced using an Illumina PE 2 × 150 by iSeq 100 i1 Reagent v2 Kit (300 cycles) on an iSeq 100 Instrument (Illumina), according to the manufacturer’s specifications. As an internal control for a low-diversity library, 10% of PhiX viral DNA was added to the sample pool. Sequencing of ITS raw data has been submitted to the NCBI SRA under the accession number PRJNA1421137. To minimise the risk of environmental contamination and reagent contamination, negative extraction controls and template-free PCR controls were included in the DNA extraction process and during library preparation, and these were sequenced alongside the test samples. All molecular procedures were carried out using standard practices to minimise the risk of contamination, including physically separated work areas before and after PCR, dedicated laboratory equipment and personal protective equipment. Single reads were removed during bioinformatic processing to minimise potential artefact or contamination-related reads.

### 2.5. Analysis of Oral Mycobiome Composition Through Bioinformatics and Statistical Methods

Sequencing reads were processed using the QIIME 2 pipeline 2024.10.1 [37]. After demultiplexing, read quality was assessed and filtered using the q2-demux plugin, and sequence denoising was performed with DADA2 [37]. Further quality filtering and length trimming were applied, and sequences were clustered into operational taxonomic units (OTUs) at a 99% identity threshold. Singletons were removed as potential artefacts or chimeric sequences. Taxonomic classification was performed using an OTU-based classifier against the UNITE dynamic database, allowing taxonomic assignment from the phylum to the species level [38].

The samples were divided into four distinct categories based on clinical phenotype and age: control_<10, PKU_<10, control_≥10, and PKU_≥10. Statistical analyses were conducted using RStudio (version 2025.09.1+401) (http://www.rstudio.com/ accesed on 10 October 2025) alongside R version 4.5.1 (developed by the Foundation for Statistical Computing, Vienna, Austria, and employed by RStudio, Inc., Boston, MA, USA) [39] to discern variations in clinical, demographic, anthropometric, and mycobiome data among matched samples. All sequencing data were processed into OTU tables, a taxonomy table, and a metadata file, and subsequently imported into the phyloseq R package (version 1.52.0, Bioconductor 3.21) [40] for further analysis. Taxonomic composition at various levels (phylum to species) was summarised and visualised using ggplot2 (version 4.0.0) [41] and ggpubr (version 0.6.3) [42]. Mean relative abundances were calculated for each taxonomic level, and group-level comparisons were visualised with horizontal bar plots. Between-group differences in the relative abundance of taxa were evaluated using Wilcoxon rank-sum tests, comparing PKU and control participants as well as age-stratified subgroups.

For alpha diversity, we calculated six diversity indices: Observed species richness, Chao1, Abundance-based Coverage Estimator (ACE), Shannon, Simpson, and Pielou’s evenness using functions from MicrobiotaProcess (version 1.21.1) [43] and vegan (version 2.7.2) [44]. Violin plots overlaid with boxplots were generated using ggplot2 and ggpubr to visualise differences across groups (PKU vs. control; age-stratified subgroups). Group comparisons were performed using the Wilcoxon rank-sum test with a significance threshold of *p* < 0.05.

We evaluated beta diversity and examined global differences in fungal community structure using Principal Coordinate Analysis (PCoA) based on Bray–Curtis dissimilarity. Ordination was performed using functions from MicrobiotaProcess and visualised using ggplot2, following Hellinger transformation of the abundance data. To further assess whether beta-diversity differences were driven by differences in OTU presence/absence rather than relative abundance, Jaccard dissimilarity was additionally calculated. PERMANOVA tests were performed using the adonis2 function from the vegan package to test for significant differences in community structure according to treatment status (PKU vs. control) and the combined age-stratified grouping variable (control_<10, PKU_<10, control_≥10, and PKU_≥10). Homogeneity of multivariate dispersion was evaluated using the betadisper function from the vegan package, followed by permutation testing, to determine whether significant PERMANOVA results reflected differences in community composition rather than unequal within-group variability.

Additionally, genus-level prevalence analyses were performed to characterise fungal occurrence patterns across groups. OTUs with abundance > 0 were considered present within a sample, and prevalence was calculated as the percentage of samples in which a given genus was detected within each subgroup. Differences in fungal prevalence between groups were assessed using Fisher’s exact test followed by Benjamini–Hochberg false discovery rate (FDR) correction.

A combination of Kruskal–Wallis tests was used for multi-group comparisons and Wilcoxon rank-sum tests for pairwise post hoc analysis to identify fungal taxa exhibiting differential abundance between groups. False discovery rate (FDR) adjustment was applied using the Benjamini–Hochberg correction, and taxa with adjusted *p* < 0.05 were considered significantly different. Adjusted *p*-values between 0.05 and 0.10 were interpreted as indicative of a statistical trend, particularly given the small sample size. To visualise group-level taxonomic distinctions, a LEfSe (Linear Discriminant Analysis Effect Size) analysis [45] was implemented using the MicrobiotaProcess package. This analysis identified taxa that significantly discriminated between the PKU and control groups based on LDA (Linear Discriminant Analysis) scores (*p* < 0.05, FDR-adjusted). To evaluate the distribution of prevalent and abundant genera, frequency–abundance plots were generated using microbiome (version 1.30.0) [46] and ggplot2. Genus-level frequency and relative abundance were computed, log_10_-transformed, and visualised separately for the PKU and control groups, highlighting potential fungal biomarkers associated with PKU status.

Predicted functional profiles were inferred using PICRUSt2 [47] and summarised at the level of KEGG Orthologs (KOs). KO abundances were log10-transformed prior to statistical analysis. Two approaches were applied: comparison between control and PKU groups (treatment), and age-stratified comparison across four subgroups (control_<10, PKU_<10, control_≥10, PKU_≥10). Differences were assessed using unpaired Student’s *t*-tests (treatment) and the Kruskal–Wallis test (multi-group) with Benjamini–Hochberg FDR correction. The top-ranked KOs based on nominal raw *p*-values were visualised for exploratory purposes. KO annotations were retrieved from the KEGG database using the KEGGREST (version 1.48.1) [48]. All analyses were performed in R (version 4.5.1) using ggplot2.

### 2.6. Analysis of Associations Between Oral Fungi and Dietary Intake Using MicrobiomeAnalyst

To explore the connection between dietary intake and fungal composition, we conducted a correlation-based pattern analysis using the MicrobiomeAnalyst 2.0 platform (https://www.microbiomeanalyst.ca/, accessed on 20 October 2025) [49]. The “Pattern Search” module was used to identify genera whose relative abundance correlated with dietary variables among all participants, encompassing both PKU individuals and healthy controls. A 15% prevalence filter was implemented to ensure robustness, retaining only those fungal taxa found in at least 15% of the samples. The analysis encompassed a range of dietary variables, including total protein (g), total fat (g), carbohydrates (g), fibre (g), saturated fatty acids (SFA, g), total sugars (g), added sugars (g), salt (g), cholesterol (mg), and Phe (mg). Additionally, it considered essential micronutrients such as vitamins (A, D, E, C, B1, B2, B3, B6, B12, folate) and minerals (magnesium, calcium, iron, zinc, selenium). Spearman’s rank correlation coefficient (|*ρ*|) was used to assess associations between dietary variables and fungal genera abundance. *p*-values were adjusted for multiple testing using the Benjamini–Hochberg FDR correction. Associations with FDR-adjusted *p* < 0.05 were considered statistically significant, whereas associations with FDR-adjusted *p* values between 0.05 and 0.15 were considered exploratory and are reported descriptively due to the pilot nature of the study. Visual outputs featured bar plots showcasing the top 25 most correlated genera for each nutrient, alongside heatmaps that illustrated taxon abundance across the four subgroups (control_<10, control_≥10, PKU_<10, PKU_≥10), to pinpoint potential diet-mycobiome associations that may vary with age or disease status.

## 3. Results

### 3.1. Cohort Description

Dietary intake data from the same population were examined in a previous study on gut microbiota [31] and re-analysed here alongside oral mycobiome profiles. The research involved 18 children (8 in the PKU group and 10 in the control group). At the beginning of the study, the mean BMI of the participants was 19.1 ± 3.7 kg/m^2^ in the PKU group and 18.9 ± 4.2 kg/m^2^ in the control group. No statistically significant differences were observed between the groups in anthropometric parameters, age, or gender distribution (Table 1).

### 3.2. Nutritional Status and Dietary Patterns in Children with PKU Compared with Controls

Participants’ nutritional status was evaluated according to the Polish reference values set by the OLA/OLAF programs, applicable to individuals aged 18 years or younger [33]. Overall, 72.2% of participants (*n* = 13) had normal body weight. Abnormal body weight was observed in 27.8% of participants *(n* = 5), including 22.2% *(n* = 4) classified as overweight and 5.6% *(n* = 1) classified as underweight. No statistically significant differences were observed between the PKU and control groups in gender distribution or nutritional status (Table 1).

Table 2 and Table 3 summarise energy, carbohydrate, and fibre intake in the control and PKU groups, respectively, in relation to the Estimated Energy Requirement (EER). These parameters were selected from the dietary records because carbohydrates represent the main substrates for bacterial and fungal biofilms.

The intake of most analysed macronutrients did not differ significantly between the groups, indicating broadly similar nutritional profiles. No significant differences were observed between the control and PKU groups in total energy intake or in the consumption of carbohydrates, dietary fibre, simple sugars, sucrose, or starch. However, lactose intake was significantly lower in the PKU group compared to controls (*p* < 0.05), which may reflect reduced consumption of dairy products.

Importantly, a statistically significant difference was also observed in Phe intake, which was lower in the PKU group than in controls (903.6 ± 970.4 mg/day vs. 3172.4 ± 1023.5 mg/day, respectively; *p* < 0.05).

Table 4 summarises the consumption of sweetened foods and beverages, the use of sweeteners, and the intake of sugar-containing products in the PKU and control groups. Eating behaviours were classified as present if they occurred at least twice per week.

Similar rates of adding sugar to food or beverages were reported in both groups (40–50%), suggesting comparable individual sweetening habits despite dietary restrictions in PKU. Similarly, high consumption of sweets and sugar-sweetened beverages was also observed in both groups, indicating that children with PKU do not completely avoid sweet products, likely due to the availability of low-Phe alternatives. The consumption of sweetened dairy products or dairy substitutes was relatively low in both groups, but was less frequent in the PKU group than in controls (12.5% vs. 30%), which is consistent with the reduced intake of lactose-containing products observed in this population. None of the children in either group reported using non-nutritive sweeteners.

### 3.3. Alpha Diversity of the Oral Mycobiome in Children with PKU Compared with Controls

**Figure 1:**
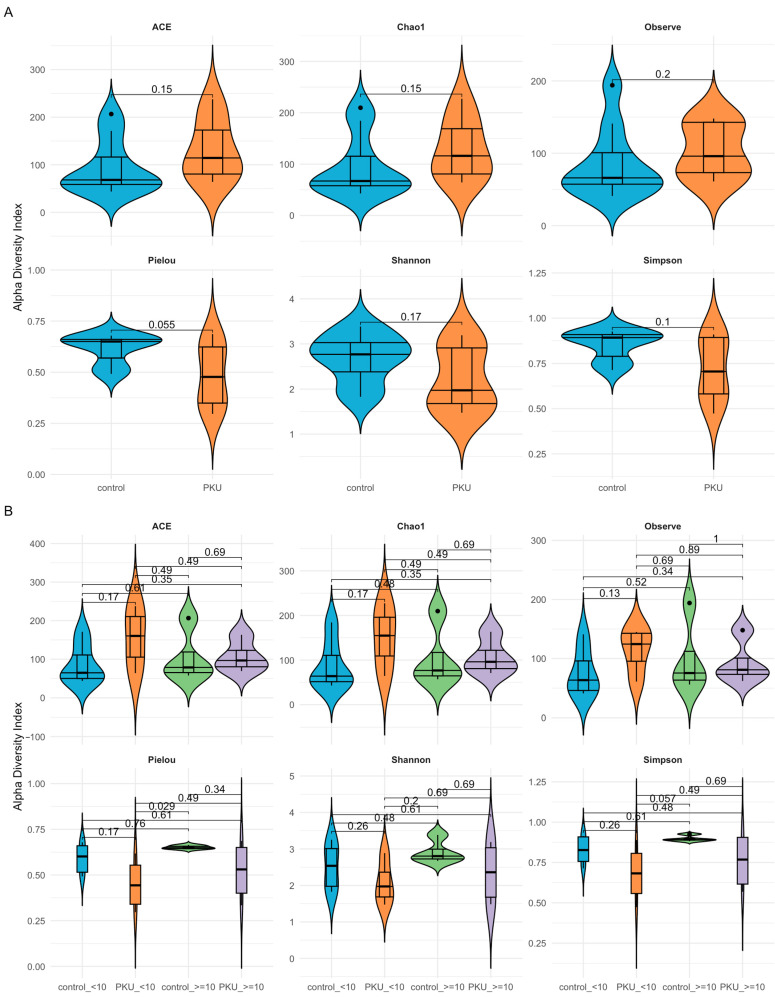
(**A**,**B**) Alpha Diversity Indices of Oral Mycobiome in PKU and Control Groups. (**A**) Comparison of six alpha diversity metrics (ACE, Chao1, Observed, Pielou, Shannon, and Simpson) between control (blue) and PKU (orange) groups. (**B**) Subgroup analysis with children stratified by age and disease status: control_< 10 (blue), PKU_< 10 (orange), control_≥10 (green), and PKU_≥10 (purple). Violin plots display the distribution and median of each index. *p*-values obtained using Wilcoxon rank-sum tests are indicated above each comparison.

Analysis of alpha diversity indices revealed no statistically significant differences in fungal richness or evenness between children with PKU and controls. Six alpha diversity metrics were assessed: ACE (*p* = 0.15), Chao1 (*p* = 0.15), observed OTUs (*p* = 0.2), Shannon (*p* = 0.17), Simpson (*p* = 0.1), and Pielou (*p* = 0.055) (Figure 1A). Although children with PKU tended to have lower values for most indices, particularly Shannon, Simpson, and Pielou evenness, none of these differences remained statistically significant after correction for multiple comparisons. Alpha diversity was further analysed across four age subgroups: control_<10, control_≥10, PKU_<10, and PKU_≥10 (Figure 1B). No significant differences were observed in any index following FDR correction. However, unadjusted *p*-values suggested possible subgroup-level patterns. Specifically, Pielou’s evenness was lower in PKU_<10 than in control_≥10 (*p* = 0.029), with a borderline trend observed for the same comparison with the Simpson index (*p* = 0.057).

### 3.4. Beta Diversity of the Oral Mycobiome in Children with PKU Compared with Controls

**Figure 2:**
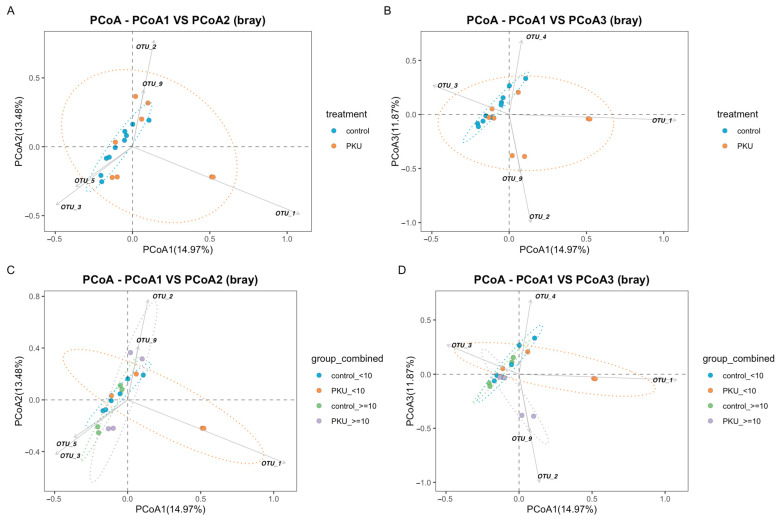
(**A**–**D**) Principal Coordinates Analysis (PCoA) of Beta Diversity of Oral Mycobiome in PKU and Control Groups Using Bray–Curtis Dissimilarity. (**A**) PCoA1 vs. PCoA2 showing partial clustering of control (blue) and PKU (orange) groups (PERMANOVA, *p* = 0.0062). (**B**) PCoA1 vs. PCoA3 for the same participants. (**C**) PCoA1 vs. PCoA2 for children stratified by age and diagnosis: control_<10 (blue), PKU < 10 (orange), control ≥ 10 (green), and PKU ≥ 10 (purple) (PERMANOVA, *p* = 0.0004). (**D**) PCoA1 vs. PCoA3 for the same four subgroups. Vectors represent the most influential OTUs contributing to sample separation; however, all OTUs were unassigned at the genus/species level. Ellipses indicate 95% confidence intervals of the group centroids.

Principal Coordinates Analysis (PCoA) was performed to assess differences in oral mycobiome structure between children with PKU and controls (Figure 2A–D). The ordination plots in Figure 2A (PCoA1 vs. PCoA2) and Figure 2B (PCoA1 vs. PCoA3) indicated partial separation of PKU and control samples along the first coordinate (PCoA1), which explained 14.97% of the total variance. However, considerable overlap between the groups was observed, suggesting substantial within-group heterogeneity and only modest differences in overall fungal community structure. The second and third axes explained 13.48% and 11.87% of the variance, respectively. Notably, the OTUs driving the separation (OTU_1, OTU_2, OTU_3, OTU_4, OTU_5 and OTU_9) could not be assigned to genus or species level using the UNITE reference database, highlighting the limitations of current fungal taxonomic annotation. Age-stratified ordination plots (Figure 2C,D) did not reveal clear clustering between younger (<10 years) and older (≥10 years) participants within either the PKU or control groups. Nevertheless, the PKU_<10 subgroup showed the greatest dispersion, suggesting higher inter-individual variability in salivary fungal profiles among younger children with PKU. This variability may be related to developmental factors and/or diet-associated exposures.

Permutational multivariate analysis of variance (PERMANOVA) was performed to formally assess differences in fungal community structure between groups. In the model including disease status (PKU vs. control) as the predictor, the oral mycobiome differed significantly between the groups (F = 1.7251, *p* = 0.0062, R^2^ = 0.097; adonis2, 9999 permutations). This indicates that group membership explained 9.7% of the variation in fungal community structure. To determine whether community structure differed across age- and diagnosis- stratified subgroups, a second model was fitted using four groups (control_<10, control_≥10, PKU_<10 and PKU_≥10). This model also revealed significant differences in beta diversity among groups (F = 1.8502, *p* = 0.0004, R^2^ = 0.284), with 28.4% of the total variation in community structure explained by the grouping factor.

Jaccard-based PERMANOVA confirmed significant differences between PKU and control groups (F = 1.5316, *p* = 0.004, R^2^ = 0.087), indicating that diagnosis explained 8.7% of the variation in presence/absence-based fungal community structure. The age-stratified model also showed significant differences among the four subgroups (F = 1.5978, *p* = 0.0002, R^2^ = 0.255), with 25.5% of the variation explained by the grouping factor. These findings suggest that the observed beta-diversity differences were driven not only by relative abundance shifts, as reflected by Bray–Curtis dissimilarity, but also by differences in OTU presence/absence across groups. Homogeneity of multivariate dispersion was assessed to determine whether the significant PERMANOVA results could be attributed to unequal within-group variability. PERMDISP analysis was not significant for Bray–Curtis dissimilarity across the four subgroups (F = 0.048, *p* = 0.986) or between treatment groups (F = 2.777, *p* = 0.117). Similarly, Jaccard-based dispersion did not differ significantly across the four subgroups (F = 0.0655, *p* = 0.979) or between treatment groups (F = 1.3493, *p* = 0.276). Therefore, the observed beta-diversity differences are unlikely to be explained by differences in group dispersion and more likely reflect genuine differences in oral fungal community composition.

To further characterise presence/absence-based differences, prevalence analysis was performed at the genus level (Tables S1 and S2). Several fungal genera showed distinct occurrence patterns between the PKU and control groups. *Hanseniaspora*, unclassified *Sporidiobolaceae*, and unclassified Agaricomycetes were detected exclusively in PKU samples, whereas *Stereum* was more frequently detected in controls (60.0%) than in PKU (12.5%). Age-stratified prevalence analysis further indicated subgroup-specific occurrence patterns, particularly within younger PKU participants, who exhibited increased prevalence of several low-abundance or unclassified taxa. Although none of these prevalence differences remained significant after FDR correction, these results support the Jaccard-based PERMANOVA findings, suggesting that oral mycobiome differences in PKU are related not only to shifts in relative abundance but also to altered fungal occurrence patterns across samples.

Collectively, these results suggest that PKU status and age appeared to be associated with differences in oral fungal community structure, with both abundance-based and presence/absence-based components contributing to the observed separation.

### 3.5. Distribution and Relative Abundance of Oral Fungi in Children with PKU Compared with Controls

**Figure 3:**
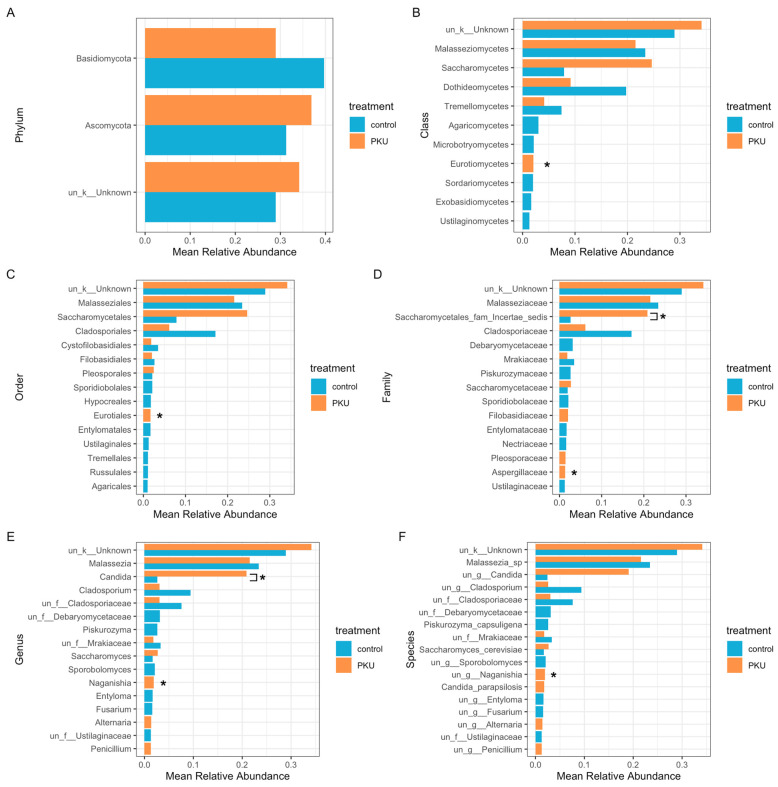
(**A**–**F**) Mean relative abundance of fungi in the oral mycobiome of the PKU group compared to the control group. Bar plots present the mean relative abundance of fungal taxa (threshold > 0.01) across six taxonomic levels: (**A**) phylum, (**B**) class, (**C**) order, (**D**) family, (**E**) genus, and (**F**) species. The x-axis indicates relative abundance, and the y-axis lists the identified taxa. Bars are coloured according to study group: blue represents the control group, and orange represents the PKU group; The asterisk (*) indicates statistically significant differences.

Taxonomic profiling revealed differences in the relative abundance of oral fungal communities between children with PKU and healthy controls across taxonomic levels (Figure 3A–F, Tables S3–S8). At the phylum level, the community was dominated by Basidiomycota, Ascomycota, and unassigned taxa, with no statistically significant differences observed between the PKU and control groups in the relative abundance of the dominant fungal phyla (*p* > 0.05) (Figure 3A, Table S3). Class-level comparisons indicated enrichment of Eurotiomycetes in PKU (2.08%) compared with controls (0.98%) (*p* = 0.027) (Figure 3B, Table S4). Saccharomycetes likewise tended to be higher in PKU (24.63%) than in controls (7.88%) (*p* = 0.10) (Figure 3B, Table S4). A similar pattern was observed at the order rank. Eurotiales were more abundant in the PKU group (1.70%) than in controls (0.86%) (*p* = 0.043) (Figure 3C, Table S5). Saccharomycetales also tended to be higher in PKU (24.63% vs. 7.88%) (*p* = 0.10) (Figure 3C, Table S5). At the family rank, several taxa distinguished the two groups. *Aspergillaceae* and Saccharomycetales_fam_Incertae_sedis showed nominal enrichment in PKU (1.35% and 20.86%, respectively) compared to controls (0.55% and 2.63%) (*p* = 0.036 and 0.011) (Figure 3D, Table S6). Conversely, *Debaryomycetaceae* tended to be less abundant in PKU (0.60%) than in controls (3.13%) (*p* = 0.068) (Figure 3D, Table S6). Consistent with these shifts, genus-level profiling revealed that *Candida* showed a nominally higher relative abundance in the PKU group (20.85%) compared with controls (2.62%) (*p* = 0.011) (Figure 3E, Table S7). *Naganishia* sp. was also detected at a higher relative abundance in PKU (1.93%) than in controls (0.00%) (*p* = 0.020), while *Penicillium* showed a borderline trend (PKU: 1.28% vs. control: 0.53%; *p* = 0.056) (Figure 3E, Table S7). At the species level, no statistically significant differences were observed between the PKU and control groups (*p* > 0.05) (Figure 3F, Table S8). Because these taxonomic comparisons were based primarily on unadjusted *p*-values and many did not remain significant after FDR correction, they should be interpreted as nominal and exploratory.

### 3.6. Oral Mycobiome Composition by Age Category and Disease

**Figure 4:**
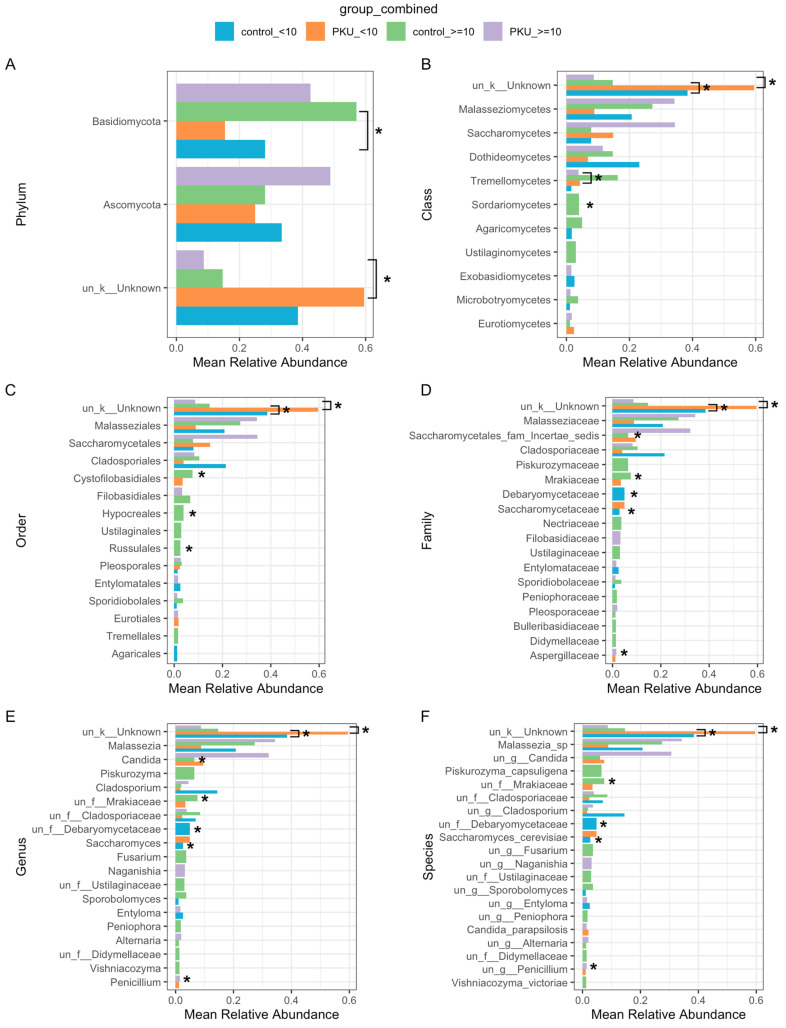
(**A**–**F**) Mean relative abundance of fungi in the oral mycobiome of the PKU and control groups, stratified by age (<10 and ≥10 years). Bar plots present the mean relative abundance of fungal taxa (threshold >0.01) across six taxonomic levels: (**A**) phylum, (**B**) class, (**C**) order, (**D**) family, (**E**) genus, and (**F**) species. The x-axis indicates relative abundance, and the y-axis lists the identified taxa. Bars are coloured according to study groups: blue for control_<10, orange for PKU_<10, green for control_≥10, and purple for PKU_≥10. The asterisk (*) indicates statistically significant differences.

Across the age-stratified subgroups, the oral fungal community was dominated by *Ascomycota* and *Basidiomycota*. *Basidiomycota* were significantly more abundant in control_≥10 than in control_<10 (57.17% vs. 28.10%, *p* = 0.038), while a trend towards higher *Ascomycota* abundance was observed in PKU_≥10 compared to control_≥10 (48.78% vs. 28.05%, *p* = 0.057) Figure 4A, Table S9). Class-level comparisons showed that *Eurotiomycetes* were higher in PKU_<10 (2.42%) than in control_<10 (0.88%, *p* = 0.038). Within the control group, *Tremellomycetes* and *Sordariomycetes* increased with age (1.61% and 0.57% in control_<10 to 16.23% and 4.06% in control_≥10; *p* = 0.0095 and 0.038). In the PKU group, *Malasseziomycetes* rose from 8.87% in PKU_<10 to 34.25% in PKU_≥10, showing a borderline trend (*p* = 0.057) (Figure 4B, Table S10). At the order level, *Eurotiales* tended to be higher in PKU_<10 (1.79%) than in control_<10 (0.85%, *p* = 0.067). In controls, *Hypocreales* were significantly more abundant in control_≥10 than in control_<10 (3.79% vs. 0.38%, *p* = 0.040), while *Russulales* showed a similar tendency (2.49% vs. 0.14%, *p* = 0.062). In addition, *Cystofilobasidiales* were detected only in control_≥10 (7.64%) (*p* = 0.029) (Figure 4C, Table S11). At the family level, *Debaryomycetaceae* were higher in control_<10 (4.87%) than in PKU_<10 (0.32%) (*p* = 0.019), whereas *Saccharomycetales_fam_Incertae_sedis* were enriched in PKU_<10 (9.59%) compared with control_<10 (0.07%) (*p* = 0.013). Additionally, *Mrakiaceae* were higher in control_≥10 (7.63%) than in PKU_ ≥10 (0.22%, *p* = 0.029), while *Aspergillaceae* were higher in PKU_≥10 (1.51%) than in control_≥10 (0.05%, *p* = 0.029) (Figure 4D, Table S12). Genus-level comparisons showed that *Candida* was higher in PKU_<10 (9.58%) than in control_<10 (0.07%) (*p* = 0.013), whereas *Saccharomyces* was higher in control_<10 (2.64%) than in control_≥10 (0.27%, *p* = 0.038) (Figure 4E, Table S13). Moreover, *Penicillium* was higher in PKU_≥10 (1.43%) than in controls (0.49%) (*p* = 0.029). Similar trends were observed at the species level. *Malassezia* sp. increased in PKU_≥10 (34.25%) compared to PKU_<10 (8.87%, *p* = 0.057), while *S. cerevisiae* was higher in control_<10 (2.64%) than in control_≥10 (0.27%, *p* = 0.038) (Figure 4F, Table S14). As these age-stratified taxonomic comparisons were exploratory and based largely on unadjusted *p*-values, they should be interpreted cautiously.

### 3.7. Group-Discriminatory Fungal Taxa and Predicted Functional Profiles (LEfSe and PICRUSt2)

**Figure 5:**
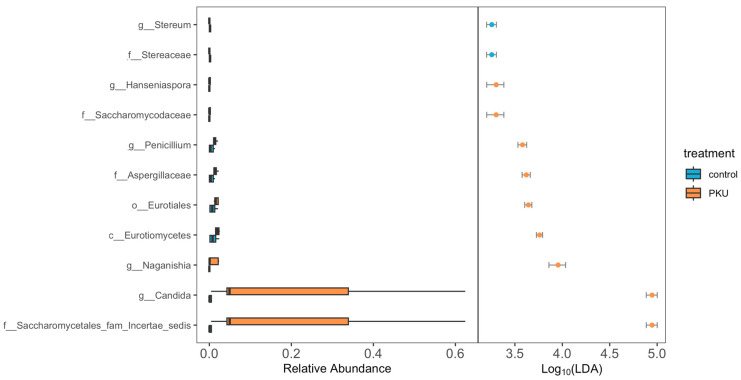
Discriminatory Oral Fungal Taxa Between PKU and Control Groups as Identified via LEfSe. The LDA scores (log_10_) for taxa that were significantly enriched in either the PKU (orange) or control (blue) group are shown in the right panel. The left panel shows the relative abundance (normalised counts) for each taxon. Only taxa with *p* < 0.05 and LDA > 3.0 are shown. Bars indicate the standard deviation of the LDA estimates based on bootstrapped intervals.

Linear Discriminant Analysis Effect Size (LEfSe) was used to identify fungal taxa that discriminated between children with PKU and healthy controls. Taxa with *p* < 0.05 and an LDA score (log_10_) > 3.0 were considered discriminatory (Figure 5, Table S15). The taxa most strongly associated with PKU were: *Saccharomycetales (Incertae sedis)* (LDA = 4.95), *Candida* (LDA = 4.95), *Naganishia* (LDA = 3.96), Eurotiomycetes (LDA = 3.76), Eurotiales (LDA = 3.64), *Aspergillaceae* (LDA = 3.62), *Penicillium* (LDA = 3.58), and *Hanseniaspora* (LDA = 3.30). In contrast, *Stereum* and *Stereaceae* were the only taxa associated with the control group (LDA = 3.26). Although all LEfSe *p*-values were <0.05, adjustment for multiple testing yielded FDR-adjusted values of 0.513 for all comparisons. Therefore, these findings should be interpreted cautiously and considered exploratory, given the limited sample size and the multiple-comparison burden. Nevertheless, the LEfSe results suggest exploratory candidate taxa that may contribute to group-level compositional differences, but should not be considered robust biomarkers.

PICRUSt2-based analysis revealed nominal differences in KEGG Orthologs (KOs) between control and PKU groups as well as across age-stratified subgroups (Figures S1 and S2, Tables S16 and S17). In the treatment comparison, the top-ranked KOs showed lower mean log10 abundances in the PKU group, with the largest differences observed for PadR family transcriptional regulator, glucokinase, and alcohol dehydrogenase; however, none remained significant after FDR correction (all adjusted *p* = 0.686). In the age-stratified analysis, several KOs reached nominal significance (*p* = 0.048–0.059), including amino acid transporter (AAT family), ribonuclease HIII, and lysine transport system proteins. Higher abundances were consistently observed in control_≥10 and PKU_≥10, whereas both <10 groups showed near-zero values. No differences remained significant after FDR correction (all adjusted *p* = 0.555). Therefore, these predicted functional profiles should be interpreted strictly as exploratory computational inferences rather than evidence of biologically validated functional alterations.

### 3.8. Selected Diet–Mycobiome Associations After FDR Correction

**Figure 6:**
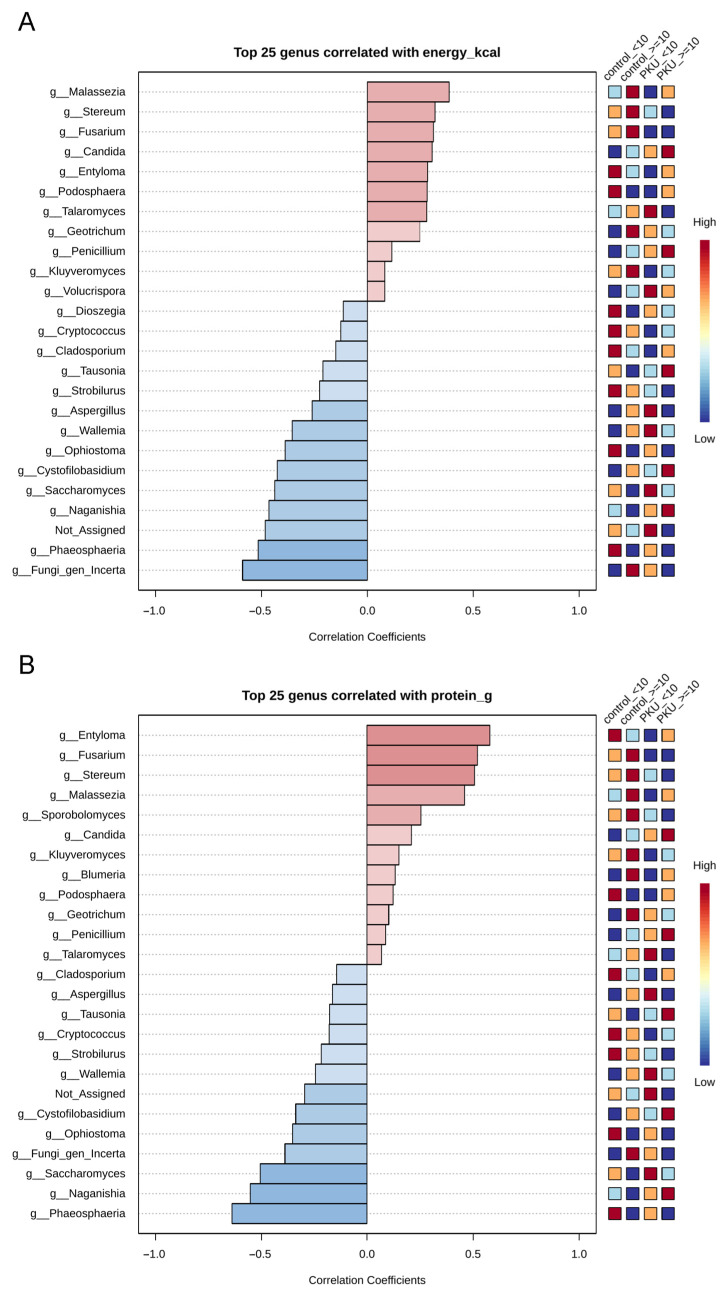

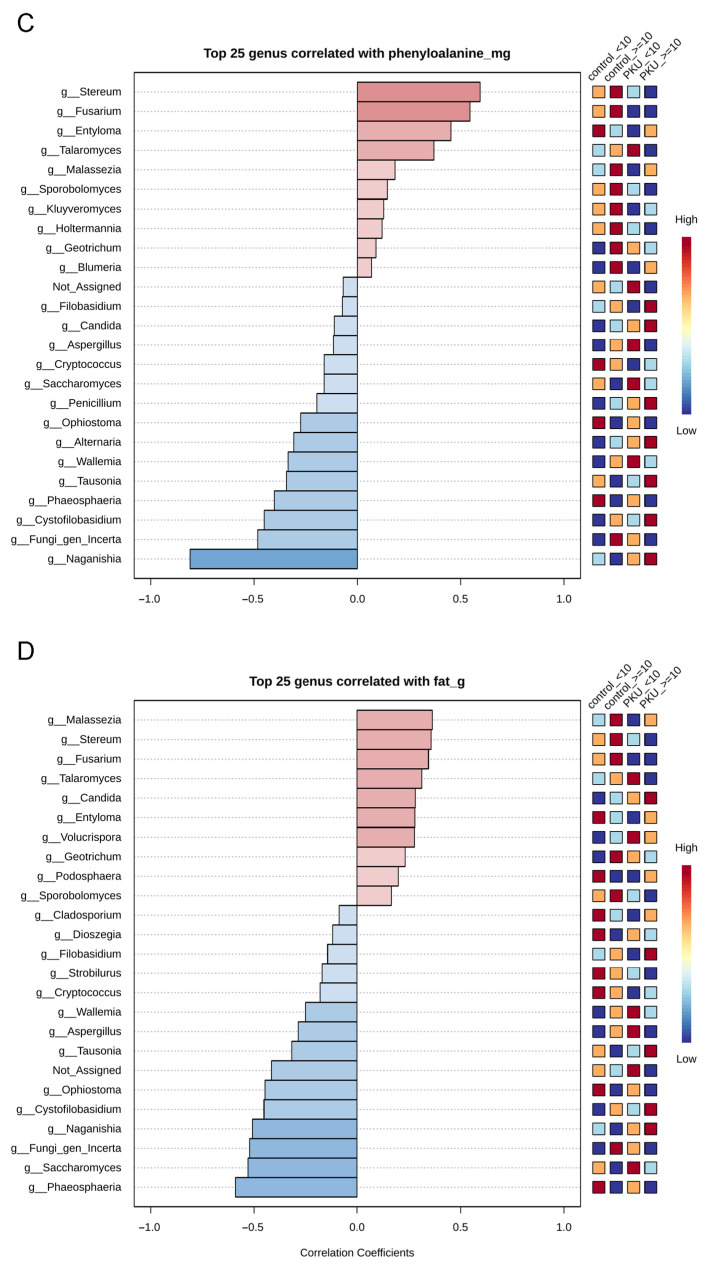

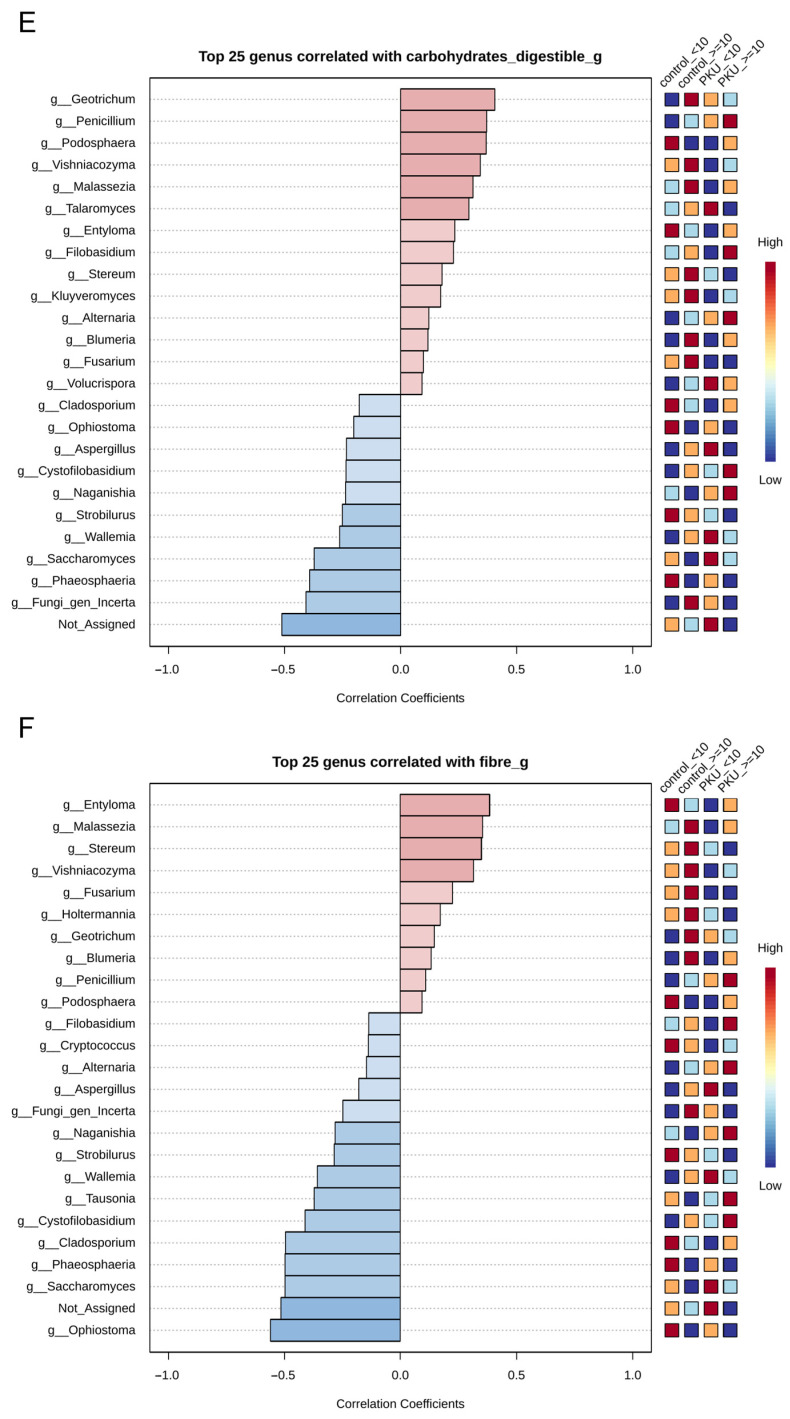
(**A**–**F**) Spearman correlations between the relative abundance of fungal genera and dietary macronutrient intake. Panels (**A**–**F**) represent correlations with (**A**) energy, (**B**) protein, (**C**) phenylalanine, (**D**) fat, (**E**) digestible carbohydrates, and (**F**) fibre. The plots display the coefficients of Spearman rank correlation, with red bars indicating positive correlations and blue bars negative ones. To the right of each plot, a heatmap depicts relative abundance across subgroups: control_<10, control_≥10, PKU_<10, and PKU_≥10. The different shades of colors indicate the relative abundance of each genus in the respective groups, ranging from low to high according to the color scale.

Several associations between dietary intake and fungal genera abundance were identified across study subgroups (Figure 6). However, after FDR correction, only a limited number of correlations remained statistically significant. The strongest association was an inverse correlation between phenylalanine (Phe) intake and *Naganishia* abundance in PKU_≥10 participants (*ρ* = −0.81, FDR = 0.0014; Figure 6C). In addition, a positive correlation was observed between n-3 fatty acid intake and *Vishniacozyma* abundance in control_≥10 participants (*ρ* = 0.72, FDR = 0.011; Figure S5E). Folate intake was inversely associated with *Saccharomyces* abundance in PKU_<10 participants (*ρ* = −0.64, FDR = 0.044; Figure S6F). Several additional nominal associations were observed for protein, saturated fatty acids, vitamins, and magnesium intake; however, these did not remain significant after multiple-testing correction and are therefore presented in the Supplementary Materials (Figures S3–S7).

## 4. Discussion

This pilot study examined the oral mycobiome of PKU patients adhering to a restrictive low-protein diet and compared it with that of healthy individuals. Furthermore, we correlated nutritional data with the mycobiome. The results indicate potential variations in oral fungal diversity and evenness between children with PKU and control subjects, especially in younger age groups.

### 4.1. Nutritional Status and Dietary Characteristics of Children with PKU

Dietary management of PKU is based on restricting phenylalanine intake through controlled natural protein consumption while ensuring adequate protein and micronutrient supply via phenylalanine-free protein substitutes [10,16]. The impact of this nutritional model on growth remains a subject of debate; however, current evidence indicates that early-diagnosed and metabolically well-controlled patients can achieve normal somatic development [50]. In the analysed cohort, no significant differences in anthropometric parameters were observed between children with PKU and controls, suggesting appropriate implementation of dietary recommendations. The intake of macronutrients, including total energy and carbohydrates, was comparable between groups, with the exception of lower lactose consumption in the PKU group, reflecting the restriction of dairy products as a source of natural protein. Although protein substitutes are typically fortified with vitamins and minerals, the dietary structure in PKU—characterised by reduced natural protein intake and reliance on specialised medical foods—may modify the overall nutrient intake profile compared with the general population [51]. Therefore, systematic nutritional assessment and individualised dietary management remain essential components of standard metabolic care.

Some studies suggest that individuals with PKU may be at risk for suboptimal status of certain B-vitamins, including riboflavin, particularly when intake of fortified medical foods is inadequate or dietary adherence is poor. Because riboflavin is essential for energy metabolism and antioxidant defence, careful nutritional monitoring is recommended in the metabolic management of PKU [52]. Niacin can be synthesised endogenously from tryptophan. In PKU, natural protein intake is restricted to limit Phe exposure; however, tryptophan is typically provided through Phe-free protein substitutes (amino acid–based medical foods), which are also commonly fortified with vitamins. Therefore, impaired niacin status is not an inherent consequence of PKU dietary treatment. Evidence regarding niacin status in PKU remains heterogeneous, and suboptimal intake may occur primarily in the context of insufficient consumption of fortified medical foods and poor dietary adherence [52]. The absence of significant differences in dietary fibre, glucose, and digestible carbohydrate intake between groups suggests that overall carbohydrate consumption patterns were comparable in children with PKU and controls. However, these findings should not be interpreted as evidence of metabolic “normalisation,” but rather as an indication of similar macronutrient distribution within the analysed dietary records.

Adequate intake of fortified protein substitutes remains essential to ensure sufficient provision of essential amino acids and micronutrients in PKU. Insufficient adherence to dietary treatment or inadequate consumption of medical foods may compromise nutritional status and, in the long term, potentially affect neurocognitive and general health outcomes. It should be noted that evidence regarding metabolic and health alterations in heterozygous *PAH* mutation carriers differs from that observed in individuals with classical PKU and cannot be directly extrapolated to this patient population [53].

### 4.2. Oral Fungal Diversity and Community Shifts in PKU

Given the pilot nature of this study and the limited sample size, the findings should be interpreted cautiously and considered hypothesis-generating. An age-stratified analysis (3–9 and 10–18 years) was carried out to account for developmental trajectories in oral mycobiome establishment, as early childhood (3–9 years) reflects primary diet-driven colonisation during tooth eruption and feeding transitions, while adolescence (10–18 years) captures puberty-related hormonal shifts influencing saliva composition and microbial niches [54]. This approach is consistent with paediatric reports describing distinct gut fungal profiles before and after the age of 10, potentially linked to dietary maturation and immune development [31].

Individuals with PKU exhibit compromised oral health, characterised by periodontitis, dental caries, and enamel hypoplasia [3,20]. Our results showed no significant differences in oral fungal alpha diversity between the PKU and control groups overall. However, a marginal reduction in Pielou evenness was evident in PKU children, potentially reflecting homogenisation from specialised low-Phe diets. In age-stratified comparisons, Pielou evenness was lower in younger PKU participants than in older controls, indicating that early-life dietary management and developmental factors may shape fungal community structure. Reduced diversity has been linked to a less resilient ecosystem, potentially affecting dental health, immunological function, and general metabolic stability [3,8]. Previous studies in PKU populations, including mixed-age cohorts (children and adults), have reported reduced oral bacterial diversity compared to controls [8].

Beta diversity analysis revealed a partial separation between PKU and controls, with stronger age-diagnosis stratification. This pattern aligns with findings that fungal communities might differ based on nutrition while exhibiting significant inter-individual variability [21,26]. This also suggests that other elements—such as host genetics, oral hygiene, and random colonisation—affect the composition of the oral mycobiome.

Additional Jaccard-based analyses demonstrated that the observed beta-diversity differences were also associated with differences in OTU occurrence, indicating that the separation between PKU and control groups was driven not only by relative abundance shifts but also by differences in the presence or absence of fungal taxa. Importantly, PERMDISP analysis was not significant, suggesting that the observed PERMANOVA results were unlikely to be explained by unequal within-group dispersion and more likely reflected genuine differences in oral fungal community composition.

### 4.3. Taxonomic Shifts Between PKU and Healthy Controls

Notable differences in fungal community structure emerged between PKU and control children. At the phylum level, Ascomycota predominated in PKU and control_<10, whereas control_≥10 showed a significantly higher proportion of Basidiomycota. This resulted in a comparatively higher Ascomycota:Basidiomycota ratio in PKU.

A high percentage of unassigned OTUs was observed, indicating the existence of novel or uncharacterized fungi; this underscores the necessity of additional sequencing and cultivation efforts to expand existing ITS reference databases [38].

At the class and order levels, PKU samples were enriched in Eurotiomycetes/Eurotiales (including *Aspergillus* and *Penicillium*) and Saccharomycetales. *Aspergillus* has been reported in the oral cavity and may be associated with both health and disease, suggesting a context-dependent role influenced by host and microbial interactions [21]. Saccharomycetales, particularly *S. cerevisiae*, are often present in both the oral and gut mycobiomes [21]. In infants, members of this order dominate throughout the first life stages, but decline as nutritional needs change [55]. In adults, *Saccharomyces* is one of the predominant genera, essential for sustaining gut homeostasis and perhaps affecting systemic immunity [55,56], and it is also commonly detected in the oral cavity [21]. *Saccharomyces* spp. ferment dietary sugars to organic acids, carbon dioxide and ethanol [57,58] and can inhibit *C. albicans* proliferation and reduce cariogenic potential in vitro, supporting a more neutral pH environment [57].

Orders such as Hypocreales and Russulales were relatively more abundant in controls. Although less studied in human oral ecosystems, Hypocreales (e.g., *Trichoderma*, *Fusarium*) are ecologically diverse and may contribute to complex trophic networks. A pilot ITS1/ITS2-based study in Polish children (*n* = 20) analysing the gut mycobiome reported higher levels of Hypocreales in controls and increased levels of *Aspergillaceae* and *Penicillium* in PKU [31], although this study was based on the gut mycobiome. In contrast, PKU fungal co-occurrence networks showed fewer unique orders, but stronger associations among Eurotiales and Saccharomycetales, suggesting a loss of breadth with tighter niche-specific interactions.

Children with PKU showed nominally higher relative abundance of several Ascomycota lineages, particularly *Saccharomycetales*: *incertae sedis* and *Aspergillaceae*. LEfSe identified *Aspergillaceae* (e.g., *Eurotiomycetes/Penicillium*), *Saccharomycodaceae* (*Hanseniaspora*) and other Ascomycota (e. g., *Saccharomycetales*: *incertae sedis* and *Candida*) as discriminant for PKU, whereas Basidiomycota taxa (e.g., *Stereaceae/Stereum*) were biomarkers for controls. Although nominally significant, these findings require cautious interpretation because several FDR-adjusted *p*-values exceeded 0.5. Consistent with previous paediatric studies, *Candida*, *Malassezia*, *Saccharomyces*, and *Cladosporium* were common constituents of the oral mycobiome [26]. The enrichment of ascomycetous yeasts in PKU likely reflects dietary patterns: patients consume a carbohydrate-rich, protein-restricted diet, promoting the proliferation of sugar-fermenting fungi [19]. Indeed, *C. albicans* and related yeasts are known to thrive on high glycaemic index diets [59].

In our dataset, *Candida* was markedly more abundant in children with PKU compared with controls, supporting evidence that simple carbohydrate intake enhances *Candida* colonisation in the oral cavity [59]. This pattern may represent a dysbiosis signature linked to metabolic or dietary disturbances in PKU. Nevertheless, because multiple oral health–related confounders were not systematically assessed, the observed *Candida* enrichment should currently be interpreted as exploratory rather than disease-specific. *Candida* species, particularly *C. albicans*, are often commensals of the oral and gut microbiota; however, they can become pathogenic under conditions such as immunosuppression or antibiotic therapy [23,60]. Their ability to adhere to host tissues and form biofilms may contribute to their persistence in the oral cavity and potential involvement in oral diseases [61].

In our study, members of the family Debaryomycetaceae tended to be more abundant in control_<10. This family, including *Debaryomyces,* is commonly detected in oral and gut mycobiomes and may contribute to mucosal homeostasis and host–microbe interactions [62,63]. *Debaryomyces hansenii*, frequently identified in oral samples, can produce xylitol from xylose, which may inhibit the growth of cariogenic bacteria such as *Streptococcus mutans*, suggesting a potential role in oral microbial balance [63].

Similarly, *Aspergillaceae*, which include filamentous fungi capable of metabolising a wide range of carbohydrates, were elevated in the PKU group. This family, predominantly represented by *Aspergillus* spp., has been detected in both healthy and caries-associated plaque in children. ITS2-based studies report *Aspergillus* at low to moderate abundance alongside *Candida* in plaques from healthy and early-childhood caries (ECC), without a consistent association with disease progression [63]. In healthy children, *Aspergillus* often co-occurs with *Penicillium* as an environmental commensal and may be more prevalent in controls than in dysbiotic states [63]. *Aspergillus* species are frequently found in food and the environment, while they may not achieve persistent colonisation, they can transiently influence mycobiome composition. Moreover, some *Aspergillus* spp. synthesise mycotoxins that may disrupt microbial equilibria and contribute to dysbiosis in oral and gastrointestinal ecosystems [64].

*Naganishia* and *Penicillium* were also significantly or marginally more prevalent in PKU. *Naganishia* is usually rare in healthy oral mycobiomes, so its higher relative abundance in PKU children may indicate dietary or ecological alterations. *Naganishia* spp. have been reported as commensals of the intestine, skin, and scalp, with some species acting as opportunistic pathogens in susceptible hosts [63]. In paediatric plaque, *Naganishia diffluens* has been identified as a core taxon, being present in 94% of samples with a mean relative abundance of around 8%, and showing no association with caries status [26]. Another ECC progression study detected *Naganishia* at low read counts, outside the top 50 taxa, and found no link with caries severity [63]. ITS2-based data therefore suggest that *Naganishia* is an environmentally stable oral resident rather than a caries-specific pathogen [26,63].

*Penicillium* species, including *P. italicum*, are widespread environmental fungi found in oral and gut communities. They may participate in complex ecological interactions with other fungi and bacteria [23]. *Penicillium* has been frequently detected in the oral cavity, including in studies of adults with periodontal disease and healthy controls, where it was among the commonly identified genera without significant differences between groups [65]. It has also been detected in the oral rinses of healthy young people alongside *Candida*, *Rhodotorula*, and *Aspergillus*, indicating transient environmental colonisation rather than a stable core role [65]. Our finding of slightly higher *Penicillium* in PKU is therefore consistent with it being a genus exposed to diet and environment, but not clearly pathogenic.

Although the overall prevalence of *Malassezia* was similar between the control and PKU groups, age-related trends emerged. Recent oral and gut studies have emphasised that *Malassezia*, while often overlooked, may be a regular member of healthy human mycobiomes [23,56]. *Malassezia* species are lipophilic yeasts that cannot ferment carbohydrates, but instead grow efficiently on lipids [66]. In the oral cavity, they may obtain lipids from saliva, gingival crevicular fluid, and dietary sources [66]. *Malassezia* thrives in slightly alkaline environments and has been linked to amino acid–fermenting bacteria, suggesting potential interkingdom metabolic cooperation. Alongside *Candida*, *Cladosporium*, and *Aspergillus*, *Malassezia* has been consistently observed in children’s saliva across caries severities [26]. Moreover, *Malassezia globosa* has been associated with caries-free dentition, implying a potentially protective or commensal role [63]. Additional work indicates that *M. restricta* and *M. arunalokei* may also be common oral residents with possible benefits for dental health [67].

Collectively, these exploratory findings suggest a possible shift in PKU towards communities with higher representation of selected Ascomycota taxa (*Candida*, *Aspergillaceae*, *Naganishia*, *Saccharomycetales: incertae sedis*), which may compromise mucosal homeostasis. This pattern is compatible with a diet- and metabolism-driven selective pressure favouring carbohydrate-adapted, fermentation-competent fungi in a protein-restricted context. Further longitudinal and functional studies are needed to disentangle cause-and-effect relationships and to characterise the specific roles of these taxa within the oral ecosystem [68].

PICRUSt2-based analysis showed only nominal differences in predicted functional profiles between control and PKU groups, with no significance after FDR correction, indicating limited evidence for disease-related functional alterations. Although some differences in predicted KO abundances were observed, these results should not be interpreted as biologically validated functional changes. PICRUSt2 provides indirect computational inference rather than direct functional measurement and was originally developed primarily for bacterial communities [8,31,47]. Its application to fungal ITS amplicon data remains limited by incomplete fungal reference databases, uncertain taxonomic assignment, and the weaker relationship between ITS-based phylogeny and fungal functional capacity. Therefore, the predicted functional profiles presented here should be regarded as exploratory and hypothesis-generating only, requiring validation using shotgun metagenomics, metatranscriptomics, metabolomics, or culture-based functional assays.

### 4.4. Diet–Mycobiome Interactions in PKU: Correlation Analysis of Nutrient Intake and Oral Fungal Composition

In this study, diet–mycobiome correlations indicated a limited number of FDR-supported associations between selected nutrients and fungal genera. Although multiple genera showed nominal associations with macronutrients, only a limited subset remained significant after FDR correction, which is consistent with the high inter-individual variability reported for paediatric plaque/salivary mycobiomes and the methodological sensitivity of ITS profiling [21,26].

The most robust finding was a strong inverse association between Phe intake and *Naganishia* in the PKU subgroup. Additionally, nominal inverse correlations were also observed between *Naganishia* and protein. Collectively, this pattern suggests that lower Phe exposure may co-occur with higher *Naganishia* abundance in this small PKU subgroup; however, this association should be interpreted cautiously because of the limited sample size, cross-sectional design, and absence of integrated bacteriome–mycobiome and clinical oral health data. Diet is known to modulate oral interkingdom interactions and biofilm formation; experimental evidence indicates that dietary substrates can reshape saliva-mediated bacterial–fungal co-aggregation and subsequent biofilm development. These data support the plausibility that diet composition can influence fungal niches even in the absence of marked shifts in alpha diversity [22,25,69].

Within lipid subcomponents, the only clearly FDR-supported association was a positive correlation between n-3 intake and *Vishniacozyma* in the control_≥10. This finding is consistent with the concept that fat quality may shape specific yeast-like taxa, potentially via lipid availability in saliva and host secretions or via lipid-sensitive interkingdom interactions. Although *Vishniacozyma* is more commonly discussed in environmental and gut datasets, diet-linked responses have been reported in other host–fungus systems, supporting the biological plausibility of such associations despite differences between oral and intestinal ecology [70].

Among B vitamins, an inverse association between folate intake and *Saccharomyces* in PKU_<10 remained significant after correction.

Overall, only a small subset of diet–mycobiome correlations remained significant after FDR correction, including *Naganishia* with Phe intake, *Vishniacozyma* with n-3 intake, and *Saccharomyces* with folate intake. These associations may indicate potential diet-related fungal patterns in children with PKU and controls; however, given the small subgroup sizes and multiple-testing burden, they should be interpreted as exploratory and hypothesis-generating. Larger longitudinal studies with functional profiling will be necessary to confirm these findings and assess their biological relevance.

### 4.5. Clinical Relevance of Oral Mycobiome Alterations in PKU

The observed fungal alterations may have important implications for oral health in PKU. Elevated levels of *Candida* and *Penicilium* in children with PKU may reflect altered oral ecological conditions associated with dietary and metabolic factors. However, because oral hygiene, dental status, fluoride exposure, salivary flow, and recent dental treatment were not comprehensively controlled in the present study, these findings should not be interpreted as direct evidence of increased caries risk or oral pathology. Instead, *Candida* enrichment should be regarded as an exploratory signal that requires validation in studies including detailed dental examination and oral health metadata. The relative reduction of health-related Basidiomycota may also reflect altered mucosal ecological balance, although its clinical relevance remains uncertain. These observations indicate that dental care in PKU may be improved by monitoring oral fungal burdens and providing guidance on both the quantity and quality of dietary carbohydrates. For example, meticulous regulation of sweetened medical foods and exploration of probiotic or pharmabiotic approaches could be considered to restore a balanced oral mycobiome.

*Saccharomyces* spp., especially *S. cerevisiae* and *S. cerevisiae* var.* boulardii*, exhibit increasingly well-documented probiotic potential, primarily described in the gastrointestinal tract [64,71]. This yeast strain has been shown to exert antimicrobial, anti-inflammatory, antioxidant and immunomodulatory effects, contributing to the maintenance of microbial homeostasis and host defence. In the oral context, in vitro data indicate that *S. cerevisiae* and *S. boulardii* can inhibit the proliferation of *C. albicans* and promote a less cariogenic, more neutral pH environment [57,72]. However, the relevance of these mechanisms in children with PKU remains speculative and requires functional validation.

The increased prevalence of *Candida* in samples from PKU patients may represent a candidate indicator of altered oral ecological conditions associated with dietary or metabolic factors. However, given the cross-sectional design, modest sample size, and lack of comprehensive oral health metadata, *Candida* should not currently be considered a validated biomarker of PKU-related oral dysbiosis or caries risk. Future studies incorporating detailed dental examination, salivary parameters, oral hygiene data, metabolic profiling, and functional mycobiome analyses will be essential to validate these findings. Such approaches may also help determine whether targeted dietary counselling, antifungal stewardship, or probiotic supplementation could have therapeutic relevance in this population.

### 4.6. Study Limitations

This preliminary investigation has several limitations. First, the cross-sectional design precludes definitive conclusions on causality; it remains unclear whether dietary changes influenced the mycobiome, whether fungal alterations affected dietary tolerance, or whether both were shaped by underlying host factors. Longitudinal designs, ideally incorporating repeated dietary assessments and time-resolved mycobiome profiling, will be required to disentangle directionality. Second, the limited sample size, particularly within age- and treatment-defined subgroups, constrains statistical power and increases the risk of both type I and type II errors. Although controls were recruited to achieve approximate group-level matching for age, sex, BMI, and nutritional status, the study was not designed as a strict matched-pairs analysis. Therefore, residual demographic heterogeneity between participants cannot be fully excluded. This limitation is reflected in the fact that many nominally significant correlations did not withstand FDR correction. Consequently, most of the associations identified here should be considered exploratory and in need of replication. An additional limitation is the incomplete characterisation of PKU metabolic control. Although all children with PKU remained under regular metabolic and dietary care, blood Phe concentrations were not available for every dietary recording period, and detailed longitudinal data regarding metabolic control, individual Phe tolerance, duration of dietary adherence, and quantitative intake of medical foods/protein substitutes were not consistently available for all participants. These variables may substantially influence nutrient exposure and oral microbial ecology and therefore represent important potential confounders that should be incorporated into future longitudinal studies. Third, a substantial proportion of ITS reads remained unclassified, highlighting the incompleteness of current reference databases for the oral mycobiome and suggesting that relevant taxa may have been overlooked in our taxonomic summaries. Further expansion of curated oral fungal databases, alongside complementary culture-based approaches, will be important to refine taxonomic resolution. Fourth, because saliva represents a relatively low-biomass microbial environment, contamination from reagents, laboratory environments, or transient environmental exposure cannot be fully excluded. To minimise this risk, negative extraction and no-template PCR controls were included throughout laboratory processing, and standard contamination-reduction procedures were applied. Nevertheless, some fungi detected in saliva may also reflect transient dietary or environmental exposure rather than stable oral colonisation. Fifth, several potentially important oral health–related confounders were not comprehensively controlled. Dental caries status, gingival inflammation, oral hygiene practices, fluoride exposure, salivary flow, recent dental treatment, mouthwash use, and dietary habits immediately before sampling may substantially influence oral fungal composition. Although all participants underwent standardised saliva collection procedures and analyses were additionally stratified by age, detailed clinical oral examinations and standardised oral health metadata were not available for all participants. Therefore, oral care-related behaviours and dental status should be considered potential residual confounders when interpreting the observed mycobiome differences, particularly *Candida* enrichment. Although saliva collection was standardised and no deviations were reported, samples were self-collected at home under parental or legal guardian supervision. Therefore, minor pre-analytical variability related to collection conditions or transport cannot be entirely excluded. Finally, the age stratification applied in this study (3 to <10 vs. 10–17.5 years) is pragmatic but inherently somewhat arbitrary, and age itself is a major driver of mycobiome variation. Future studies with larger cohorts should explore finer-grained age categories and include age as a continuous covariate in multivariable models. Taken together, these limitations underscore that our conclusions should be interpreted as hypothesis-generating. Nevertheless, the consistent signals involving Phe, fat quality, and selected vitamins provide a rationale for more comprehensive, longitudinal, and mechanistic studies of diet–mycobiome interactions in children with PKU. Accordingly, the present findings should be interpreted as exploratory and hypothesis-generating rather than definitive evidence of PKU-specific oral mycobiome alterations.

### 4.7. Future Research Perspectives

Further work should validate these preliminary findings in larger, longitudinal cohorts of children with PKU to assess the temporal stability of the oral mycobiome and its responsiveness to dietary changes, age, and metabolic control. Multi-kingdom studies integrating bacterial, viral, and fungal data could clarify how PKU diets reshape interkingdom networks across oral and gut niches. Functional approaches such as shotgun metagenomics, metatranscriptomics and metabolomics are needed to determine whether the taxa enriched in PKU are associated with pathways relevant to carbohydrate and amino-acid metabolism, mucosal immunity, and inflammation, in line with oral and gut data. Priorities include establishing links between oral fungal profiles and Phe levels, nutritional status and inflammatory markers, as well as comparing oral and gut mycobiome signatures within the same patients. Intervention studies should test whether dietary modifications (e.g., lower glycaemic index, optimised fat quality, improved micronutrient status) or mycobiome-directed strategies (probiotics, next-generation Phe-degrading probiotics, or carefully selected antifungal/probiotic combinations) can beneficially modulate oral communities without compromising metabolic control. Finally, improved characterisation of unassigned OTUs by whole-genome or long-read sequencing may reveal previously unrecognised oral fungi relevant to PKU. This research underscores the need for continuous monitoring and personalised dietary strategies to deal with the distinct metabolic requirements of individuals with PKU while supporting a good quality of life.

## 5. Conclusions

This pilot study indicates that children with PKU exhibit measurable compositional differences in their oral mycobiome compared with healthy peers. Although alpha diversity was broadly comparable between groups, beta diversity differed significantly by diagnosis and was further influenced by age, supporting disease- and development-related community shifts. Taxonomically, PKU samples showed nominally higher representation of selected environmentally or diet-associated taxa, including *Candida*, *Naganishia* and *Aspergillaceae*, although these findings should be interpreted cautiously due to multiple-testing limitations. Importantly, diet–mycobiome analyses suggested that specific nutrient dimensions may be more informative than overall digestible carbohydrate intake, as highlighted by the strong inverse associations between Phe intake and *Naganishia*, and additional nutrient-linked signals involving n-3 fatty acids and folate in age-stratified analyses. Together, these findings support the hypothesis that PKU dietary therapy and metabolic context shape oral fungal ecology and may be associated with altered oral ecological patterns in a population considered vulnerable to dental complications. The predicted functional analyses presented here should be considered preliminary computational inferences rather than validated functional evidence. Integrating oral mycobiome assessment with dietary evaluation and age-specific dental surveillance may help to refine personalised nutritional strategies for PKU; however, clinical translation will require confirmation. Future studies should also integrate longitudinal metabolic control variables, including blood Phe concentrations, dietary adherence, Phe tolerance, and medical food use, to better define the relationship between PKU management and oral fungal ecology. Larger longitudinal cohorts, rigorous control of oral health status and hygiene-related confounders and functional profiling (e.g., metabolomics and/or metatranscriptomics) are warranted to clarify mechanisms, assess stability over time and test whether targeted dietary optimisation or mycobiome-directed interventions can improve oral health outcomes in paediatric PKU. However, given the limited sample size, these findings should be considered preliminary and hypothesis-generating until validated in larger cohorts.

## Figures and Tables

**Table 1:** Comparison of demographic and anthropometric parameters and nutritional status between control and PKU children.

No	Gender	Age	Body Mass (kg)	Height (m)	BMI (kg/m^2^)	Nutritional Status
Control group						
C1	Male	17.0	74.0	1.71	25.3	Overweight
C2	Female	12.8	58.0	1.62	22.1	Norm
C3	Female	8.6	25.0	1.32	14.3	Underweight
C4	Male	3.0	14.0	0.98	14.6	Norm
C5	Female	5.0	22.0	1.16	16.3	Norm
C6	Female	17.0	67.0	1.66	24.3	Norm
C7	Male	13.0	52.0	1.53	22.2	Overweight
C8	Male	6.4	24.0	1.26	15.1	Norm
C9	Male	9.5	38.0	1.42	18.8	Norm
C10	Female	3.4	16.0	1.00	16.0	Norm
AVG	–	9.6 ^A^ ± 5.2	39.0 ^A^ ± 22.1	1.4 ^A^ ± 0.3	18.9 ^A^ ± 4.2	–
PKU group						
PKU1	Male	17.0	70.0	1.70	24.2	Overweight
PKU2	Female	3.0	17.3	1.00	17.3	Norm
PKU4	Male	13.0	64.0	1.59	25.3	Overweight
PKU5	Male	6.0	25.0	1.24	16.3	Norm
PKU6	Female	17.5	50.6	1.64	19.0	Norm
PKU7	Male	3.0	14.0	0.89	17.7	Norm
PKU9	Female	12.7	37.0	1.56	15.2	Norm
PKU10	Female	9.8	33.4	1.36	17.8	Norm
AVG	–	10.2 ^A^ ± 5.8	38.9 ^A^ ± 20.9	1.4 ^A^ ± 0.3	19.1 ^A^ ± 3.7	–

^A^ Mean scores with different letters in the same column are significantly different at *p* < 0.05 (Student’s *t*-test or Mann-Whitney U test). Abbreviations: AVG—average; BMI—Body Mass Index; Compliance with the Standard (%)—percentage of the recommended intake achieved; Norm—intake within the recommended range; Above—intake above the recommended range; Under—intake below the recommended range.

**Table 2:** Energy, carbohydrate and fibre consumption patterns and compliance with dietary recommendations in control children.

No	Energy (kcal)			Carbohydrates (g)			Dietary Fiber (g)		
	Mean Daily Intake	EER	Compliance with the Standard (%)	Mean Daily Intake	45–65% of EER (g)	Compliance with the Standard	Mean Daily Intake	AI (g)	Compliance with the Standard
C1	2635.9	2933	89.9	326.3	330.0–476.6	Norm	35.9	21	Enough intake
C2	2343.8	2111	111.0	361.1	237.5–343.0	Norm	35.8	19	Enough intake
C3	1854.4	1686	110.0	267.3	189.7–274.0	Norm	20.2	16	Enough intake
C4	1205.2	1163	103.6	165.6	130.8–189.0	Norm	8.3	10	Deficiency
C5	1255.8	1419	88.5	187.2	159.6–230.6	Norm	14.3	14	Enough intake
C6	2027.4	2255	89.9	263.1	253.7–366.4	Norm	33.0	21	Enough intake
C7	1519.8	2384	63.8	211.7	268.2–387.4	Under	19.2	19	Enough intake
C8	2158.8	1620	133.3	288.5	182.3–263.3	Norm	19.9	14	Enough intake
C9	1746.6	1934	90.3	266.2	217.6–314.3	Norm	13.4	16	Deficiency
C10	1260.8	1088	115.9	175.8	122.4–176.8	Norm	19.9	10	Enough intake
AVG	1800.9 ^A^ ± 494.4	–	–	251.3 ^A^ ± 65.3	–	–	22.0 ^A^ ± 9.7	–	–
No	Simply sugars (g)			Saccharose (g)		Lactose (g)		Starch (g)	
	Mean Daily Intake	10% of EER	Compliance with the Standard (%)	Mean Daily Intake		Mean Daily Intake		Mean Daily Intake	
C1	60.5	73.3	Norm	56.8		2.1		168.5	
C2	78.6	52.8	Above	60.2		18.0		169.0	
C3	59.3	42.2	Above	41.5		16.3		113.4	
C4	38.0	29.1	Above	17.0		7.1		72.8	
C5	39.2	35.5	Above	30.0		7.9		95.8	
C6	42.2	56.4	Norm	33.3		8.9		136.1	
C7	44.0	59.6	Norm	34.0		10.0		127.2	
C8	95.2	40.5	Above	74.4		20.8		108.3	
C9	42.8	48.4	Norm	37.6		5.2		152.5	
C10	47.8	27.2	Above	36.2		11.6		61.3	
AVG	54.8 ^A^ ± 19.0	**–**	**–**	42.1 ^A^ ± 16.9		10.8 ^A^ ± 5.9		120.5 ^A^ ± 37.6	

^A^ Mean scores with different letters in the same column in Table 2 and Table 3 are significantly different at *p* < 0.05 (Student’s *t*-test). Abbreviations: AI—Adequate Intake; AVG—Average; EER—Estimated Energy Requirement; Compliance with the Standard (%)—percentage of the recommended intake achieved; Norm—intake within the recommended range; Above—intake above the recommended range; Under—intake below the recommended range: C1–C10—control samples.

**Table 3:** Energy and carbohydrate consumption patterns and compliance with dietary recommendations in PKU children.

No	Energy (kcal)			Carbohydrates (g)			Dietary Fiber (g)		
	Mean Daily Intake	EER	Compliance with the Standard (%)	Mean Daily Intake	45–65% of EER (g)	Compliance with the Standard	Mean Daily Intake	AI (g)	Compliance with the Standard
PKU1	2149.4	2933	136.5	272.2	330.0–476.6	Under	21.2	21	Enough intake
PKU2	1232.8	1088	88.3	213.2	122.4–176.8	Above	10.5	10	Enough intake
PKU4	2752.9	2384	86.6	422.2	268.2–387.4	Norm	30.4	19	Enough intake
PKU5	1843.6	1620	87.9	271.8	182.3–263.3	Norm	17.7	14	Enough intake
PKU6	1854.5	2255	121.6	329.5	253.7–366.4	Norm	35.3	21	Enough intake
PKU7	1045.9	1096	104.8	194.8	123.3–178.1	Norm	13.5	10	Enough intake
PKU9	1654.4	2016	121.9	267.2	226.8–327.6	Norm	18.7	19	Enough intake
PKU10	1205.1	1790	148.5	192.6	201.4–290.9	Norm	23.8	16	Enough intake
AVG	1717.3 ^A^ ± 566.4	–	–	270.4 ^A^ ± 77.1	–	–	21.4 ^A^ ± 8.3	–	–
No	Simply sugars (g)			Saccharose (g)		Lactose (g)		Starch (g)	
	Mean Daily Intake	10% of EER	Compliance with the Standard (%)	Mean Daily Intake		Mean Daily Intake		Mean Daily Intake	
PKU1	69.1	73.3	Norm	61.2		7.7		152.9	
PKU2	95.8	27.2	Above	91.7		0.3		96.3	
PKU4	48.4	59.6	Norm	48.2		0.3		105.3	
PKU5	67.0	40.5	Above	61.0		4.0		125.1	
PKU6	57.8	56.4	Norm	56.6		0.7		152.8	
PKU7	42.8	27.4	Above	41.6		1.1		100.1	
PKU9	19.9	50.4	Norm	15.9		3.8		161.1	
PKU10	27.8	44.8	Norm	17.0		3.9		97.8	
AVG	53.6 ^A^ ± 24.4	–	–	49.2 ^A^ ± 24.9		2.7 ^B^ ± 2.6		123.9 ^A^ ± 27.8	

Values marked with different superscript letters within the same column differ significantly at *p* < 0.05, according to Student’s *t*-test. Abbreviations: EER—Estimated Energy Requirement; AI—Adequate Intake; Compliance with the Standard (%)—percentage of the recommended intake achieved; Norm—intake within the recommended range; Above—intake above the recommended range; Under—intake below the recommended range.

**Table 4:** Patterns of sweetened food and beverage consumption in the control and PKU groups.

	Sweetened Foods and Beverages	Sweeteners	Sugary Drinks	Sweets	Sweetened Dairy Products or Substitutes
No	Control group				
C1	X		X	X	
C2			X	X	
C3				X	X
C4					X
C5				X	
C6	X		X	X	
C7				X	
C8				X	X
C9	X			X	
C10	X			X	
No	PKU group				
PKU1	X		X	X	
PKU2	X			X	
PKU4	X		X		X
PKU5			X	X	
PKU6			X	X	
PKU7	X				
PKU9					
PKU10				X	

X is used as a marker/indication.

## Data Availability

The original ITS sequencing raw data presented in the study are openly available in NCBI SRA under accession number PRJNA1421137 (https://dataview.ncbi.nlm.nih.gov/object/PRJNA1421137?reviewer=5q9cf8m02ptt53p522v68c1drn).

## Supplementary Materials

The following supporting information can be downloaded at: https://www.mdpi.com/article/10.3390/nu18111764/s1. Figure S1. Differences in predicted KEGG Ortholog abundances between control and PKU groups; Figure S2. Differences in predicted KEGG Ortholog abundances across age-stratified subgroups; Figure S3. (A–F) Spearman correlation between carbohydrate, salt components and the relative abundance of the top 25 fungal genera across study subgroups. Figure S4. (A–E) Spearman correlation between micronutrient intake and the relative abundance of the top 25 fungal genera across study subgroups. Figure S5. (A–E) Spearman correlation between dietary components and the relative abundance of the top 25 fungal genera across study subgroups. Figure S6 (A–F) Spearman correlations between the relative abundance of fungal genera and intake of dietary fat components. Figure S7 (A–F) Spearman correlations between the relative abundance of the top 25 fungal genera and B-vitamin intake in four study subgroups. Table S1—Presence/absence prevalence of oral fungal genera between control and PKU groups. Table S2—Presence/absence prevalence of oral fungal genera across age- and diagnosis-stratified subgroups. Table S3. Oral Fungal Phylum Abundance and Statistical Comparison Between Control and PKU Groups; Table S4. Oral Fungal Class Abundance and Statistical Comparison Between Control and PKU Groups; Table S5. Oral Fungal Order Abundance and Statistical Comparison Between Control and PKU Groups; Table S6. Oral Fungal Family Abundance and Statistical Comparison Between Control and PKU Groups; Table S7. Oral Fungal Genus Abundance and Statistical Comparison Between Control and PKU Groups; Table S8. Oral Fungal Species Abundance and Statistical Comparison Between Control and PKU Groups; Table S9. Oral Fungal Phylum Abundance and Statistical Comparison Between Control and PKU Groups (control_<10 vs. PKU_<10; control_≥10 vs. PKU_≥10; control_<10 vs. control_≥10; PKU_<10 vs. PKU_≥10); Table S10. Oral Fungal Class Abundance and Statistical Comparison Between Control and PKU Groups (control_<10 vs. PKU_<10; control_≥10 vs. PKU_≥10; control_<10 vs. control_≥10; PKU_<10 vs. PKU_≥10); Table S11. Oral Fungal Order Abundance and Statistical Comparison Between Control and PKU Groups (control_<10 vs. PKU_<10; control_≥10 vs. PKU_≥10; control_<10 vs. control_≥10; PKU_<10 vs. PKU_≥10); Table S12. Oral Fungal Family Abundance and Statistical Comparison Between Control and PKU Groups (control_<10 vs. PKU_<10; control_≥10 vs. PKU_≥10; control_<10 vs. control_≥10; PKU_<10 vs. PKU_≥10); Table S13. Oral Fungal Genus Abundance and Statistical Comparison Between Control and PKU Groups (control_<10 vs. PKU_<10; control_≥10 vs. PKU_≥10; control_<10 vs. control_≥10; PKU_<10 vs. PKU_≥10); Table S14. Oral Fungal Species Abundance and Statistical Comparison Between Control and PKU Groups (control_<10 vs. PKU_<10; control_≥10 vs. PKU_≥10; control_<10 vs. control_≥10; PKU_<10 vs. PKU_≥10); Table S15. Differential Abundance of Fungal Taxa Between Control and PKU Groups Identified by LEfSe. Table S16. Top 10 KEGG Orthologs differentiating control and PKU groups; Table S17. Top 10 KEGG Orthologs across age-stratified subgroups.
