# Supplementary material for: Oral Mycobiome Alterations in Children with Phenylketonuria: Associations with Dietary Intake and Metabolic Context—A Pilot Study
Source: Nutrients. 2026 May 30;18(11):1764. doi: 10.3390/nu18111764 (PMC13259183; doi:10.3390/nu18111764)
Supplement: Supplementary file 1 [file nutrients-18-01764-s001.zip › nutrients-4322726-supplementary/Supplementary materials/nutrients-4322726-supplementary materials-confirmed.pdf]

Supplementary materials

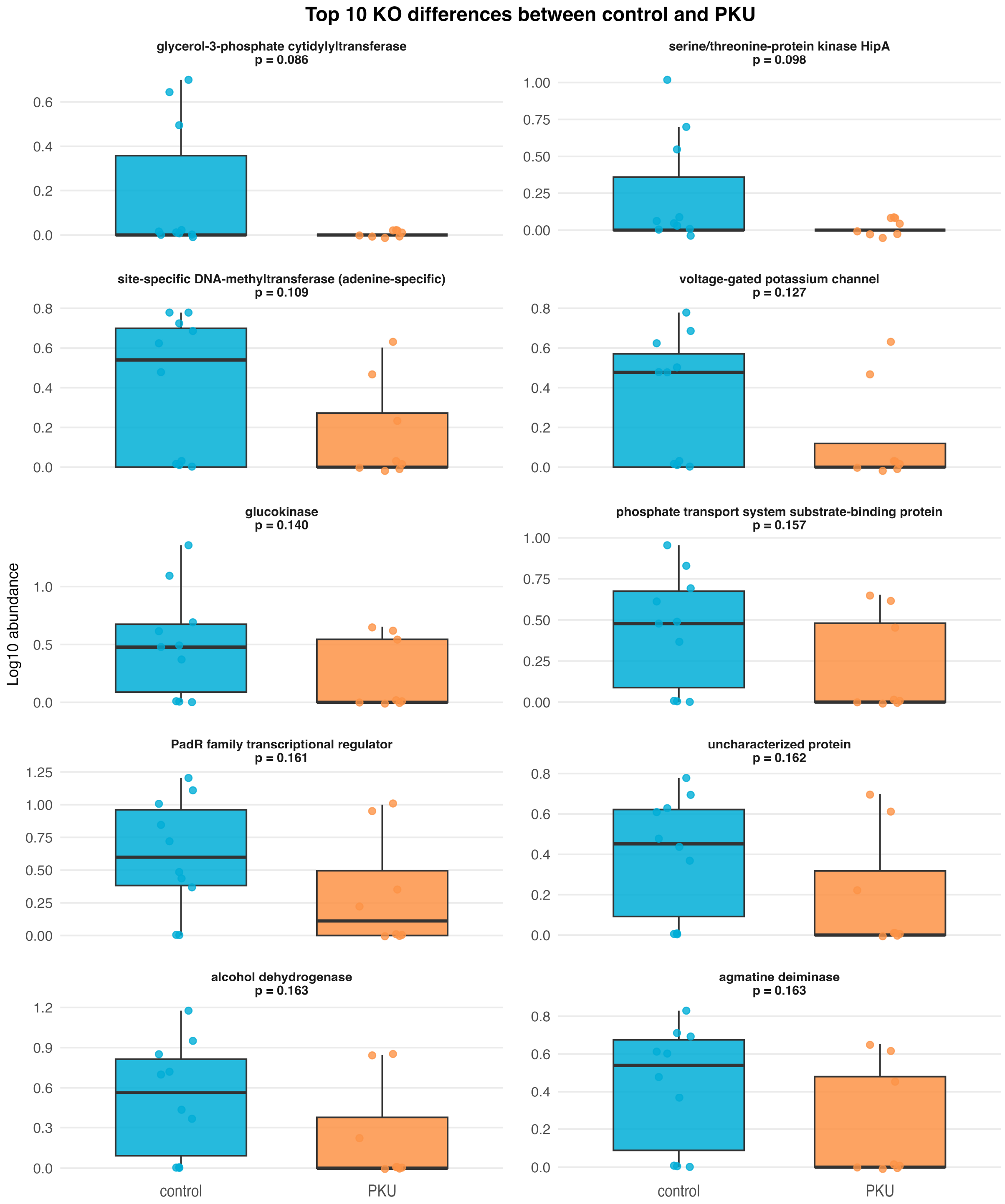

Figure S1. Differences in predicted KEGG Ortholog abundances between control and PKU groups. Boxplots showing log10-transformed abundances of the top 10 KEGG Orthologs (KOs) ranked by raw *p*-value from unpaired Student's t-tests comparing control and PKU groups. Each point represents an individual sample. Boxes indicate the median and interquartile range. Raw *p*-values are shown within panel titles. None of the differences remained statistically significant after FDR correction.

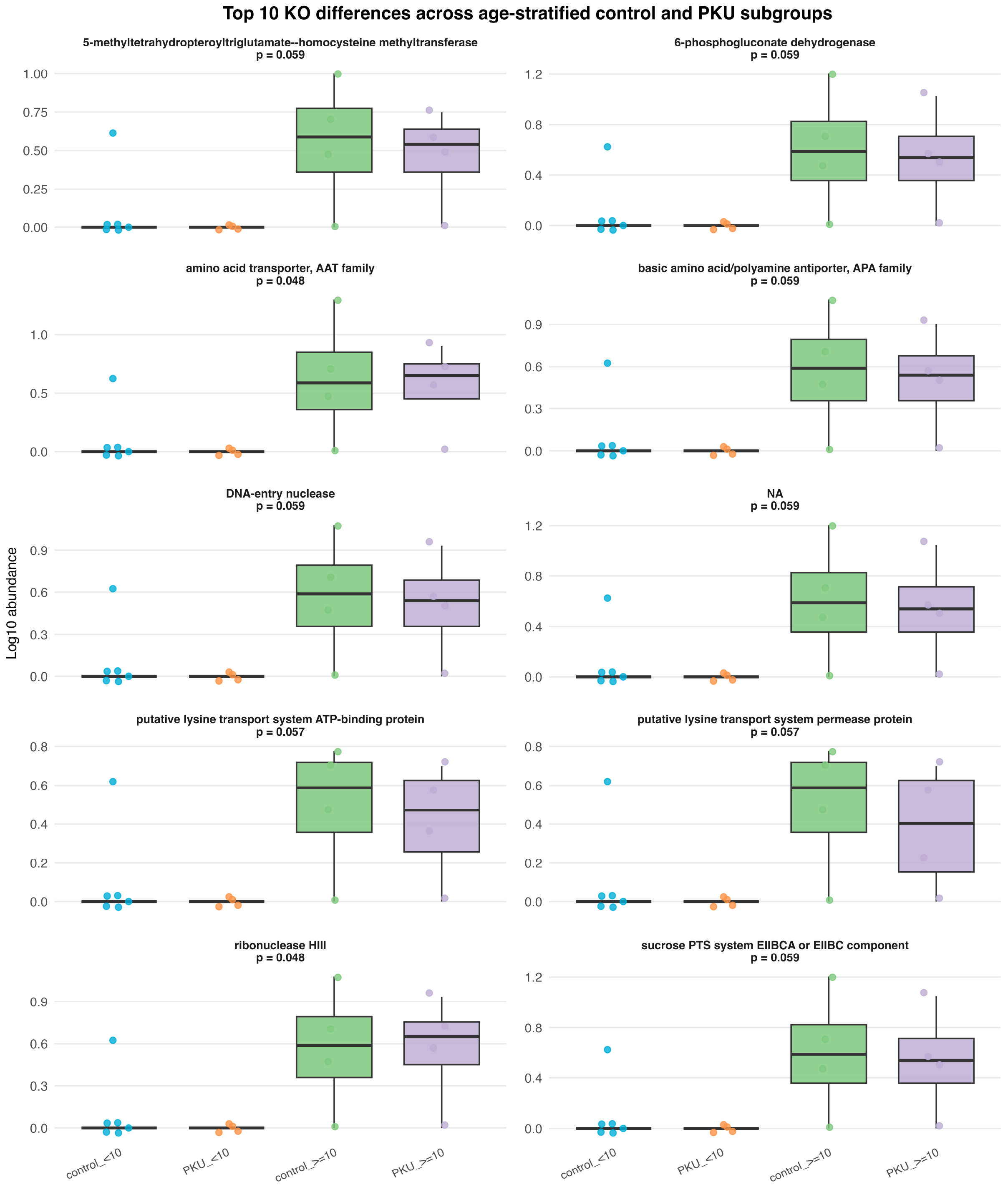

Figure S2. Differences in predicted KEGG Ortholog abundances across age-stratified subgroups. Boxplots showing log10-transformed abundances of the top 10 KEGG Orthologs (KOs) ranked by raw *p*-value from Kruskal–Wallis tests across four subgroups (control_<10, PKU_<10, control_≥10, PKU_≥10). Each point represents an individual sample. Boxes indicate the median and interquartile range. Raw *p*-values are shown within panel titles. None of the differences remained statistically significant after FDR correction.

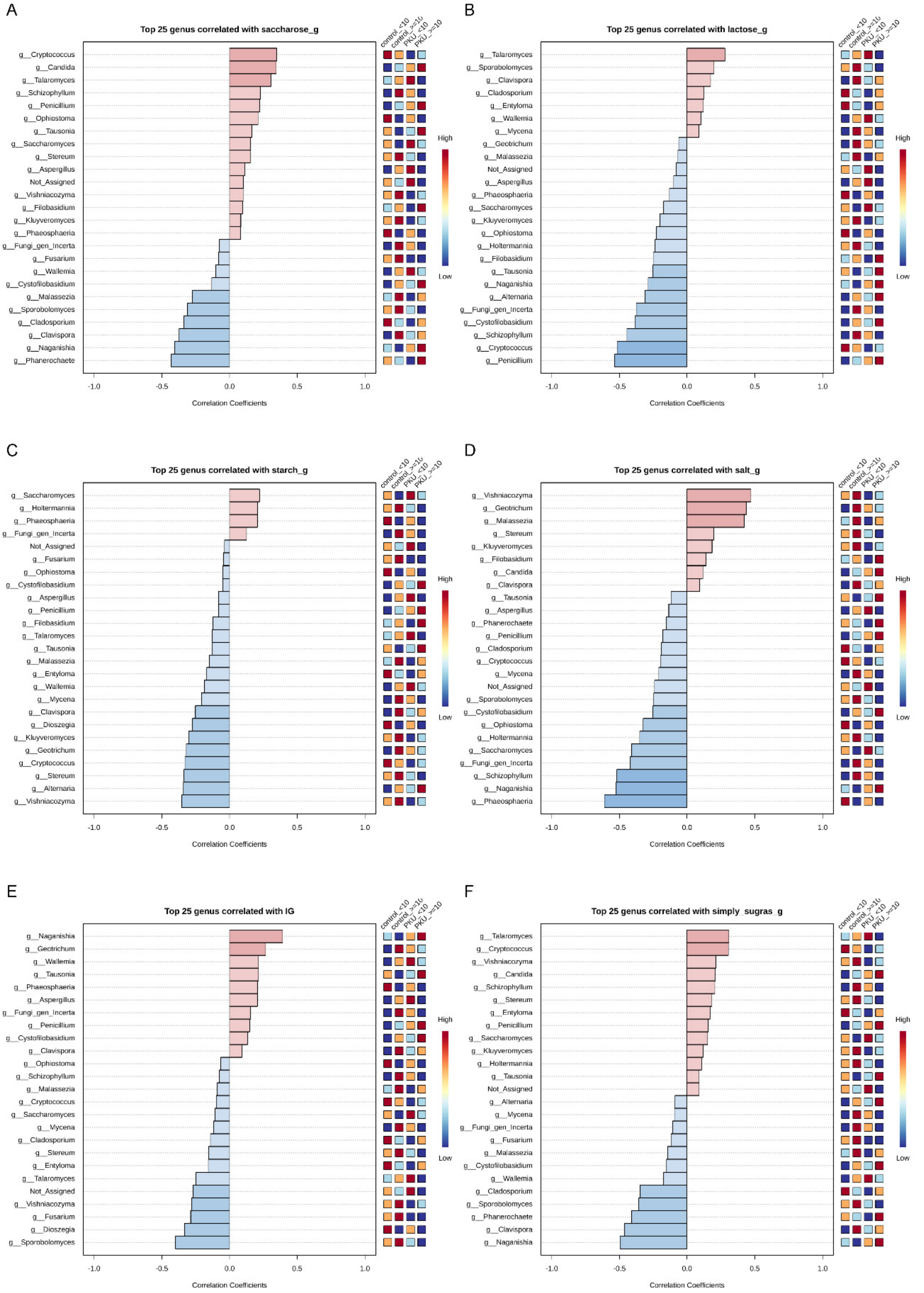

Figure S3. (A-F) Spearman correlation between carbohydrate, salt components and the relative abundance of the top 25 fungal genera across study subgroups. Panels A–F display correlation matrices for: (**A**) saccharose, (**B**) lactose, (**C**) starch,

(**D**) salt, (**E**) glycemic index (GI), and (**F**) simple sugars. The plots display the coefficients of Spearman rank correlation, with red bars indicating positive correlations and blue bars negative ones. To the right of each plot, a heatmap depicts relative abundance across subgroups: control_<10, control_≥10, PKU_<10, and PKU_≥10.

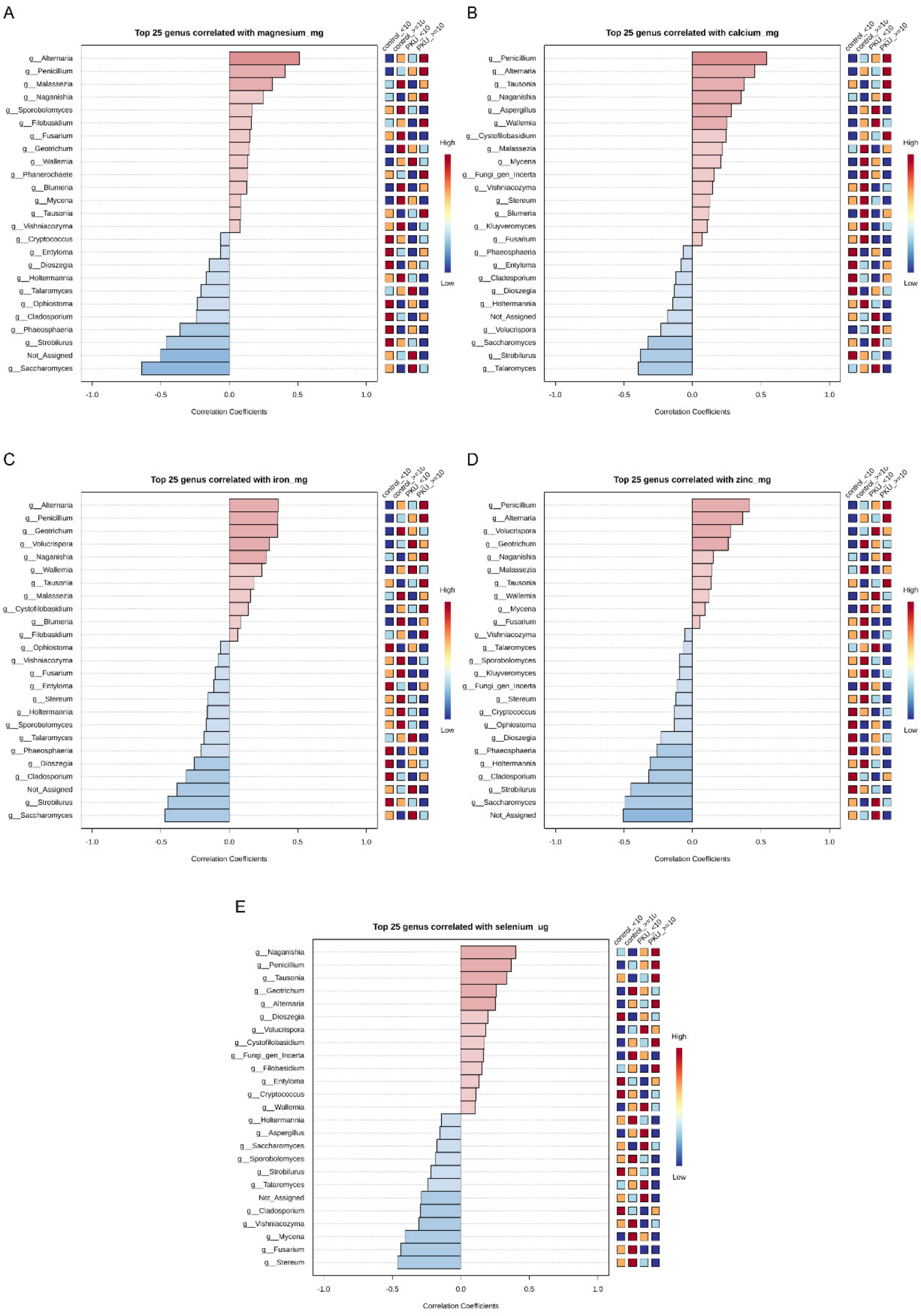

Figure S4. **(A-E)** Spearman correlation between micronutrient intake and the relative abundance of the top 25 fungal genera across study subgroups. Panels **A–F** display correlation matrices for: (**A**) magnesium, (**B**) calcium, (**C**) iron, (**D**) zinc,

(**E**) selenium. The plots display the coefficients of Spearman rank correlation, with red bars indicating positive correlations and blue bars negative ones. To the right of each plot, a heatmap depicts relative abundance across subgroups: control_<10, control_≥10, PKU_<10, and PKU_≥10.

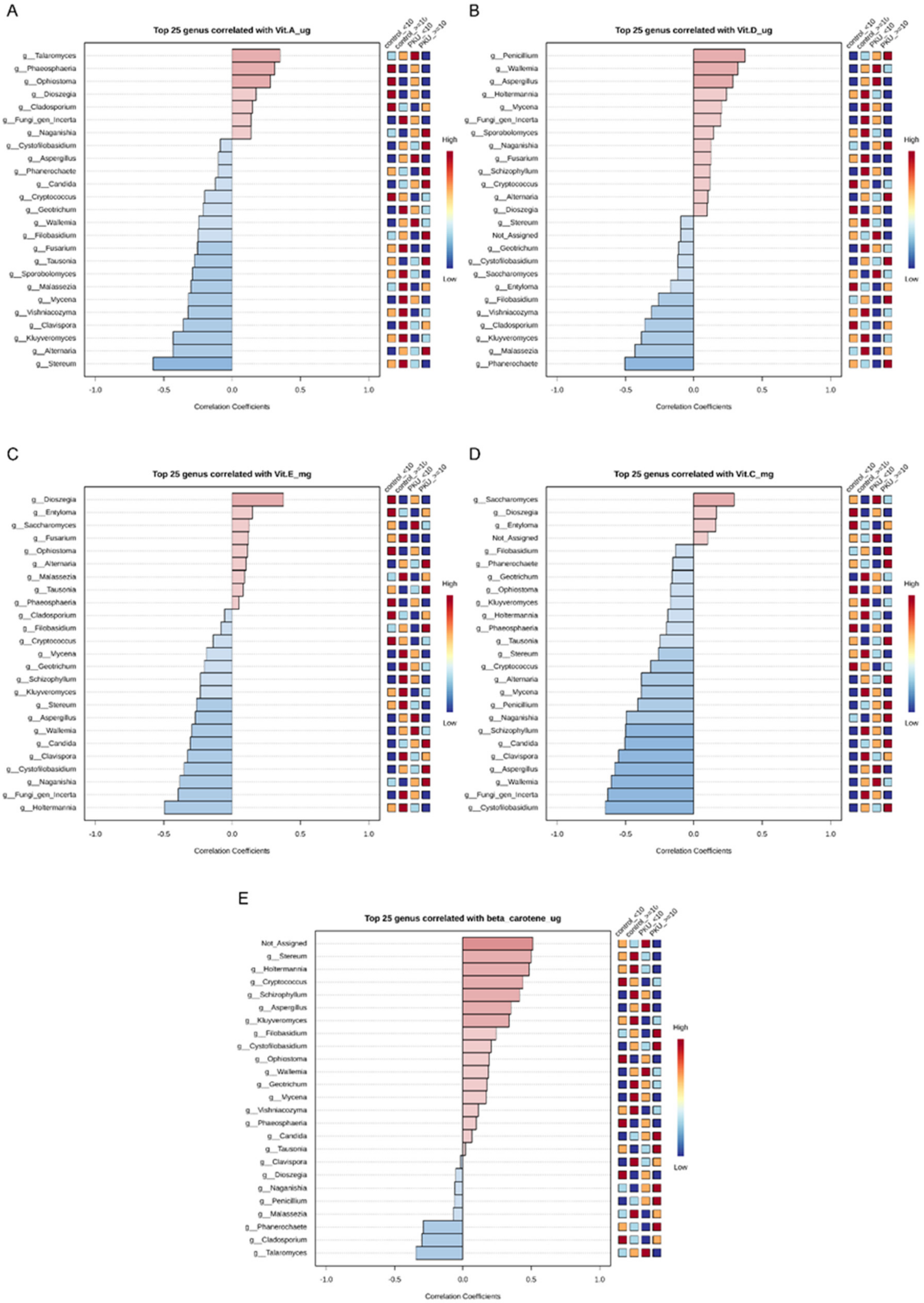

Figure S5. (A–E) Spearman correlation between dietary components and the relative abundance of the top 25 fungal genera across study subgroups. Panels A–F display correlation matrices for: (**A**) Vit. A, (**B**) Vit. D, (**C**) Vit. E, (**D**) Vit. C, (**E**)

beta carotene. The plots display the coefficients of Spearman rank correlation, with red bars indicating positive correlations and blue bars negative ones. To the right of each plot, a heatmap depicts relative abundance across subgroups: control_<10, control_≥10, PKU_<10, and PKU_≥10.

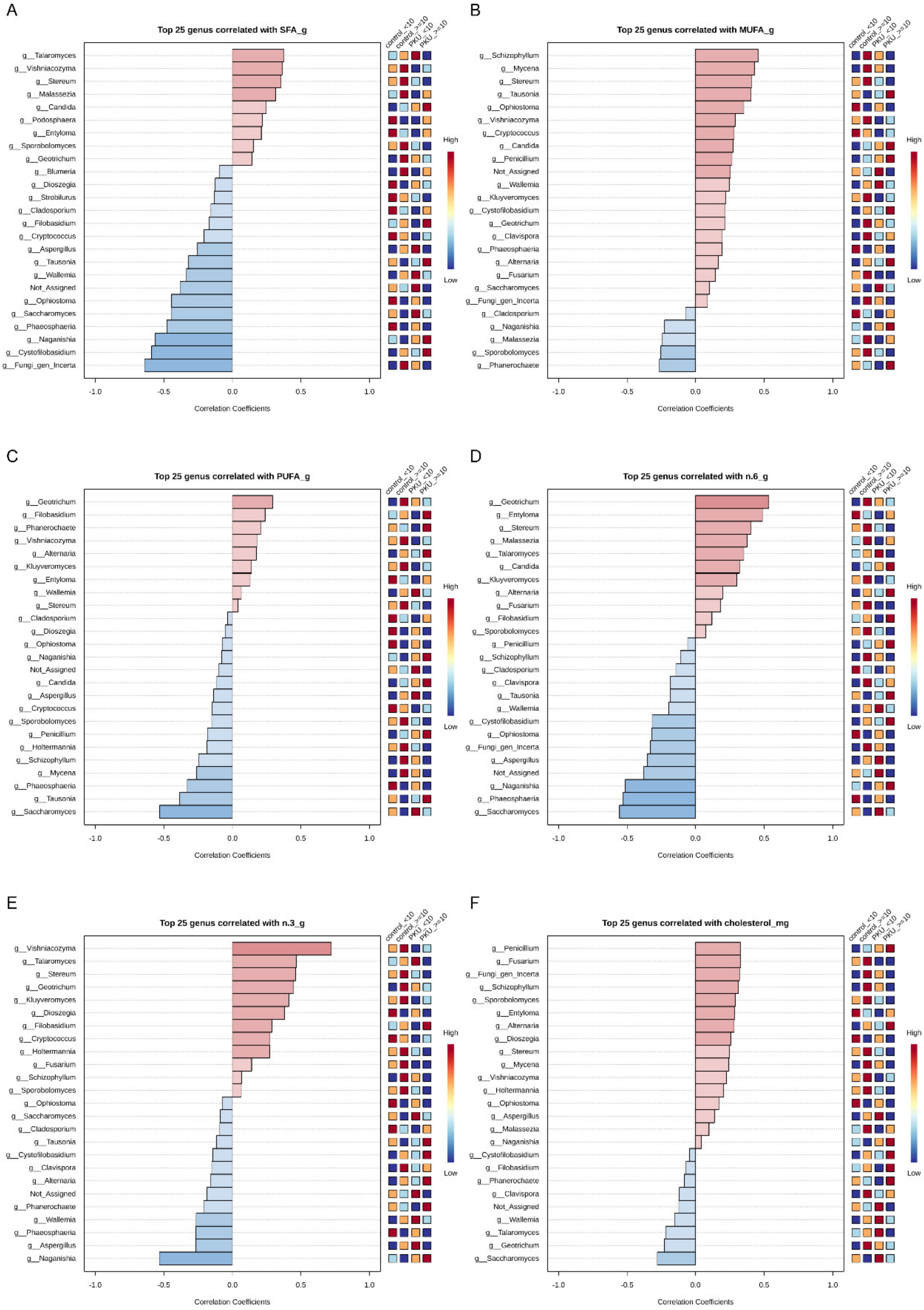

Figure S6 (A-F) Spearman correlations between the relative abundance of fungal genera and intake of dietary fat components. Panels **A–F** represent correlations

with (**A**) saturated fatty acids (SFA), (**B**) monounsaturated fatty acids (MUFA), (**C**) polyunsaturated fatty acids (PUFA), (**D**) n-6, (**E**) n-3 fatty acids, and (**F**) cholesterol. The plots display the coefficients of Spearman rank correlation, with red bars indicating positive correlations and blue bars negative ones. To the right of each plot, a heatmap depicts relative abundance across subgroups: control_<10, control_≥10, PKU_<10, and PKU_≥10.

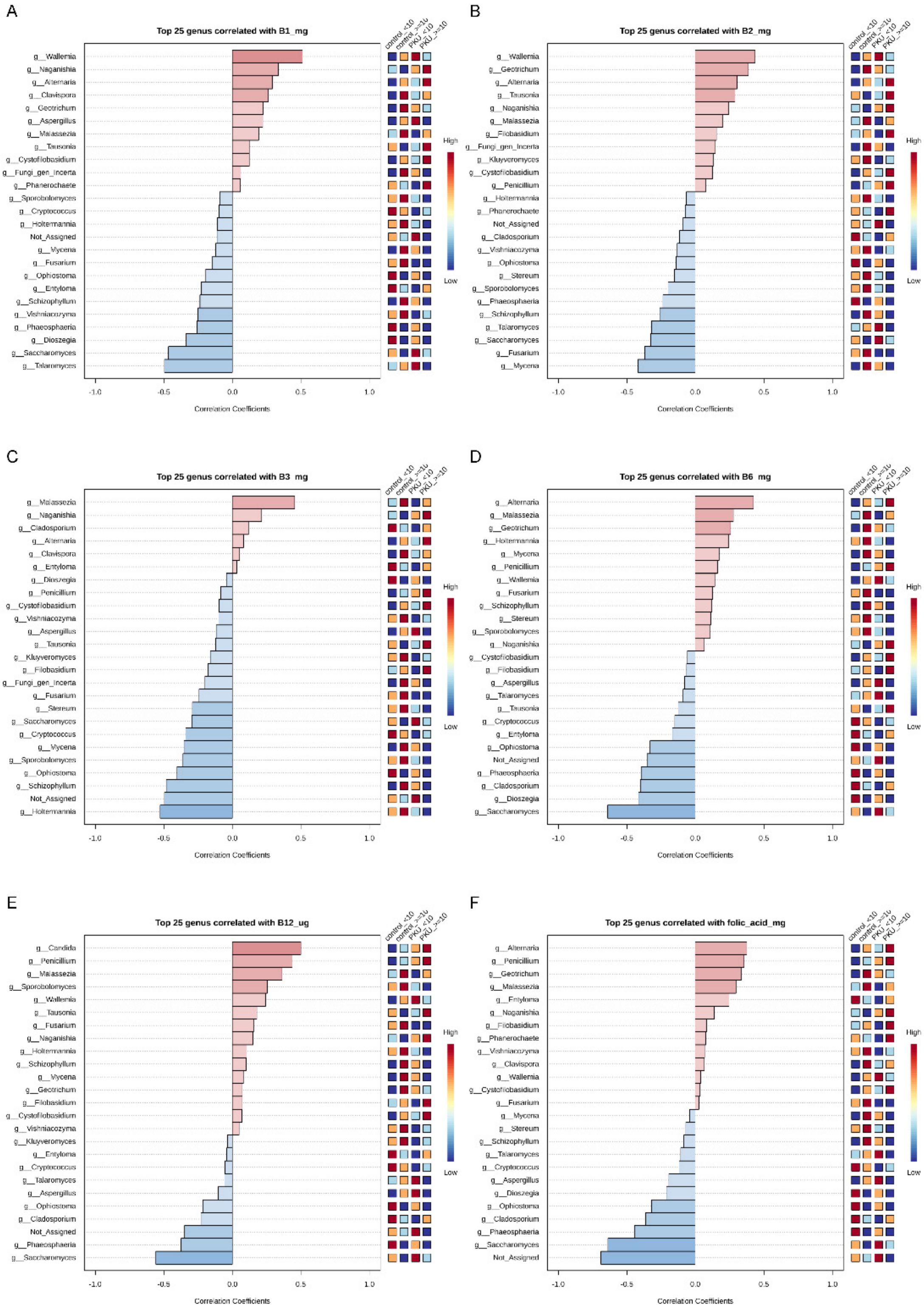

Figure S7 (A-F) Spearman correlations between the relative abundance of the top 25 fungal genera and B-vitamin intake in four study subgroups. Panels A–F display correlation matrices for: (A) B1, (B) B2, (C) B3, (D) B6, (E) B12, and (F) folic acids. The plots display the coefficients of Spearman rank correlation, with red bars indicating positive correlations and blue bars negative ones. To the right of each plot, a heatmap depicts relative abundance across subgroups: control_<10, control_≥10, PKU_<10, and PKU_≥10.
